# Drivers and influence of social conformity on decision making in human-AI teams

**DOI:** 10.1038/s41598-026-43042-5

**Published:** 2026-03-13

**Authors:** Huixin Zhong, Jack McKinlay, Jinha Yoon, Rashi Bagri, Eamonn O’Neill, Janina A. Hoffmann

**Affiliations:** 1 School of Smart Governance, Renmin University of China, Suzhou, 215123 China; 2 Department of Computer Science, UKRI Centre for Doctoral Training in Accountable, Responsible and Transparent AI, University of Bath, Bath, BA2 7AY UK; 3 Department of Psychology, University of Bath, Bath, BA2 7AY UK

**Keywords:** Decision-making, Human-AI teams, Social conformity, Informational influence, Normative influence, Mathematics and computing, Psychology, Psychology

## Abstract

Advances in AI are transforming decision making teams. A fundamental open question is whether AI agents elicit social conformity effects comparable to human peers. If so, a critical challenge is determining whether the established drivers of social conformity in human teams—informational influence (the need to gain information from others) and normative influence (the need to gain approval or avoid punishment from others)—also transfer to AI teammates. In two studies, we investigated informational and normative influence in mixed human–AI teams using a medical information cascade paradigm. Participants received private evidence about a medical case and public advice from one or more AI or human advisors. Study 1 explored how participants weighted this public advice compared to their private information in scenarios where private and public information were equally accurate. Study 2 varied the accuracy of the private and public information. Results showed a distinct contrast between the two mechanisms. Humans and AI exerted a similar level of informational influence in both Study 1 and Study 2, and participants systematically under-weighted advice from both human and AI advisors relative to a Bayesian computational benchmark when source accuracies varied in Study 2. However, normative influence differed by advisor identity. In Study 1, participants placed significantly more weight on human advice than on equally reliable AI advice, indicating that AI lacks the normative pull inherent to human peers. Crucially, Study 2 revealed that this preference is fragile. We found that participants did not rely on a single heuristic, but instead attempted to integrate majority consensus with variable advisor accuracy. This complex processing eliminated the normative bias toward humans, and participants systematically under-weighted advice from both humans and AIs relative to the Bayesian computational reference. In addition, we also found an egocentric bias in both studies: participants in both studies weighed their private information significantly more than public advice. Theoretically, these findings suggest that differential reliance on AI versus human advice stems from the absence of normative influence rather than a lack of informational trust. Practically, the results highlight a design risk: presenting heterogeneous accuracy cues may increase cognitive load, leading to suboptimal advice integration.

**Supplementary Information:**

The online version contains supplementary material available at 10.1038/s41598-026-43042-5.

## Introduction

Advances in AI technology are shifting the paradigm of decision making from human-only to collaborative human-AI teams, particularly in high stakes domains such as medical decision making^1^. Concurrently, to enhance system robustness and integrate diverse expertise, advanced AI architectures increasingly task multiple agents with specific sub-problems^2–6^. While the development of human-AI teams aims to leverage complementary strengths of the two parties for higher decision quality^7,8^, they introduce a new layer of social complexity: do these emerging AI teammates elicit the same social conformity observed in human groups? In human teams, individuals frequently align with the majority despite contradictory evidence^9^. While group consensus can sometimes improve accuracy^10^, it can also lead to collective error^11^. Yet, whether this social conformity extends to AI teammates remains a critical open question. Most existing research on the conformity to AI focuses on how individuals conform to a single AI^12,13^ or on whether individuals are more inclined to conform to a single AI or to a single human advisor^14^. We extend this work by exploring how individuals conform to advice from mixed teams composed of multiple humans and AI.

However, establishing whether social conformity occurs in human-AI teams is only the first step. A more critical theoretical challenge lies in distinguishing the underlying mechanisms. In human teams, social conformity typically stems from two distinct drivers^15^: the desire to reduce uncertainty by seeking information (*informational influence*) or the desire to gain social acceptance (*normative influence*). Previous research in human-AI teams has predominantly investigated whether robots^16,17^ or virtual agents^18,19^ can elicit *normative social influence*. Crucially, these studies often rely on tasks with obvious correct answers, where reliance on others is purely a social act. This focus overlooks a critical reality: AI is typically deployed in environments characterized by high uncertainty. In such contexts, advice serves a primary function of providing valid data to reduce error, thereby generating strong *informational influence*. Yet, despite its importance in real world decision making, the role of informational influence and how it interacts with normative pressures has rarely been explicitly considered in human-AI conformity research. In this paper, we address this gap by extending the classical theory of informational and normative influence^15^ to human-AI teams. Adopting a behavioural economics perspective, we apply a social influence model to simultaneously quantify the degree to which both informational and normative drivers shape social conformity. Beyond establishing these baseline drivers, we further investigate their robustness in ecologically valid settings. Our second study independently varies the accuracy of information sources, enabling us to examine how individuals trade off advice from multiple, less accurate AI agents against single, highly accurate humans.

First, we provide controlled empirical evidence distinguishing the specific mechanisms underlying differential conformity with respect to human versus AI advisors. In our study, we operationalise informational influence as adjustment driven by the perceived accuracy of the information, consistent with the fundamental epistemic motivation to reduce uncertainty and optimise decision outcomes by treating advice as valid diagnostic evidence^20^. Conversely, we operationalise normative influence as the extra weight assigned to an advice source even when two sources are equally valid without overt social pressure cues. This captures the internalised dimension of normative influence that operates implicitly, automatically and independently of overt social coercion^21,22^. We demonstrate that, much like in human teams, social conformity in human-AI teams is shaped by both informational and normative drivers. Our results reveal a critical dissociation: when decision accuracy is held constant, the informational influence exerted by AI agents is comparable to that of humans. In contrast, the normative influence is significantly weaker in human-AI teams compared to human-only teams. This suggests that the baseline preference for human advisors stems primarily from the normative influence inherent to human interaction, rather than a lack of informational trust in AI. However, we find that this human normative advantage is fragile. When information validity from humans and AIs varied and participants attempted to integrate distinct accuracy cues with majority consensus, the normative bias toward humans disappeared. Secondly, we employ a Bayesian updating model to quantify and dissociate the distinct weights participants assign to different information sources. Unlike traditional measures of conformity, this computational approach allows us to precisely estimate the weight given to private information versus advice from multiple human and AI agents. Practically, applying this diagnostic to mixed teams (Study 2) reveals a critical design trade-off regarding information cues: we find that simply presenting explicit accuracy cues alongside majority consensus does not automatically improve calibration. Instead, it leads to a systematic discounting of advice relative to the statistical baseline, which we interpret as a sign that such combinations may increase cognitive demand rather than clarity. This highlights that for mixed human-AI teams, effective interface design must balance information richness with cognitive load to prevent the sub-proportional integration of valid advice.

## Related work

### Theoretical mechanisms of social conformity

Social conformity, the act of adjusting one’s behaviour to match others^23^, is theoretically underpinned by two distinct drivers of decision making in human teams: *informational* and *normative* influence^15^. Informational influence arises when individuals treat others’ inputs, behaviour or advice as valid evidence, driven by the desire to be accurate^23^. In contrast, normative influence stems from the desire to gain social approval or avoid sanctions^24^.

While early conceptual work treated these mechanisms independently^25,26^, they are often intertwined in the decision making process. Research on information cascades demonstrates that social conformity is not merely a social ritual but can be a rational strategy to integrate public information (advice from others) with private knowledge (information available only to the decision maker)^20,27^. However, a persistent challenge in this domain is empirically disentangling the two drivers. In high stakes environments such as medicine, an assistant may follow a senior physician’s advice due to recognized expertise (informational influence) or power (normative influence)^22^. To resolve this ambiguity, Schöbel et al.^22^ introduced a probabilistic cascade paradigm that separates the two drivers by quantifying how participants weigh “public” advice against “private” signals. This approach enables precise measurement: informational influence is observed when advice increases decision accuracy and confidence, whereas normative influence is detected when advice is weighted disproportionately heavily relative to its statistical validity.

### Social influence in human-agent interaction

A critical theoretical question is whether this dual-process framework extends beyond human-human interaction to contexts involving non-human agents, such as computers, robots and avatars. In digital environments, it is well established that the communication medium and the degree of social presence can significantly modulate conformity^28,29^. Within this context, existing literature presents a divided picture. Prior findings reveals a divergence: while agents reliably exert *informational influence*, their capacity to exert *normative influence* is inconsistent and highly conditional. Research indicates that computers and robots can trigger strong informational conformity, particularly when tasks are difficult or time-constrained, as users treat the agent as a superior analytical tool^16,30^. Recent work extends this to text-based agents, showing that LLMs can trigger conformity specifically when they provide rationales, suggesting informational influence^31^. However, the normative influence is often missing. Studies utilizing avatars^18,19^ or robots^17^ in classic line-judgment tasks generally found that these agents failed to elicit normative conformity unless they possessed strong anthropomorphic cues, such as human-like appearance or gaze. This suggests a theoretical limitation in prior work: observed conformity to agents often relied on visual anthropomorphism (simulating a human) rather than the agent’s intrinsic status as a teammate. This leaves a gap regarding AI systems which may be increasingly abstract and disembodied yet tasked with high stakes collaboration.

### Research gap and objectives

Most previous research has focused on the question of whether single robots, avatars or anthropomorphic computer agents can elicit normative influence. Studies explicitly investigating social conformity to AI agents have predominantly considered interactions between one human and one AI rather than advice from mixed teams composed of multiple humans and AI^12,13,32^. Focusing solely on dyadic interactions or groups comprised entirely of AIs presents a critical limitation: it precludes determining the relative strength of AI influence compared to that of humans. We add to this work by investigating how mixed human-AI teams exert social influence. Unlike single-agent settings, mixed teams create a comparative context where the user must weigh the normative pressure from a human teammate against the advice of an AI. This allows us to directly measure the social influence and whether the presence of human peers alters reliance on AI.

Furthermore, studies distinguishing informational from normative influence largely suggest that robots and anthropomorphic agents elicit conformity via informational rather than normative influence. Yet, these studies have several methodological limitations that we address. First, regarding the agent representation, previous work typically induced normative influence by manipulating visual appearance or non-verbal social cues, potentially confounding normative influence with anthropomorphism. In real-world scenarios, however, AI is rarely depicted with human-like features and is more often presented as a numerical prediction or a flag highlighting a preferred option. To understand the influence of advice from AI in such scenarios–and to isolate influence from mere appearance–it is necessary to minimize anthropomorphism by using a very abstract AI representation. Secondly, regarding the task nature, previous work has typically operationalised informational influence by manipulating task ambiguity (high *vs* low) in tasks that require a clear cut right or wrong answer. Yet, AI is increasingly deployed to support decision making in complex scenarios with high uncertainty, where neither one’s own information nor the advice offered definitively provides the “correct” answer. Very few studies have assessed how humans deal with such probabilistic information from non-human agents. Using an information cascade paradigm allows us to systematically vary the degree of uncertainty via the evidence supporting one option. Methodologically, we advance previous research by applying a Bayesian updating model as a measurement tool. This allows us to go beyond observing simple conformity rates to quantify and disentangle the specific degrees of normative and informational influence in these complex, probabilistic environments.

In summary, we still lack a good understanding of what drives social conformity in AI, informational or normative influence. Therefore, our first study investigated the following questions: (i) does social conformity influence decision making in human-AI teams?; and (ii) are there differences regarding the two drivers of social conformity (informational and normative) between human only teams and human-AI teams?

## Study one: differentiating drivers of social conformity

### Study design: decision making in human-AI teams

We adopted a modified information cascade paradigm, which was originally proposed by Schöbel and colleagues^22^. They were able to separate normative from informational influence in a medical information cascade paradigm^22^. Participants imagined themselves as assistant physicians who had to diagnose patients with one of two possible illnesses, *A* or *B*, and also had to rate their confidence in their decisions. To make this diagnosis, participants received a piece of private information, i.e. a symptom the patient had, and up to three pieces of public information in the form of diagnoses from “colleagues”. Each piece of information, public and private, was equally valid and its validity was defined as the conditional probability, *p*(*a*|*A*), that a symptom or prior diagnosis pointing towards *a* was presented given that the patient had disease *A*. Using Bayes’ theorem, the posterior probability $p(A|n_a,n_b)$ of disease *A* given $n_a$, the number of symptoms and diagnoses presented that suggest disease *A*, and $n_b$, the number of symptoms and diagnoses presented that suggest disease *B*, can be calculated as formula 1:

1 $$\begin{aligned} p(A|n_a,n_b) = \frac{p(n_a,n_b|A) p(A)}{p(n_a,n_b|A) p(A) + p(n_a,n_b|B) p(B)} \end{aligned}$$

Schöbel’s paradigm has four advantages for our study. First, their medical decision making scenario allows us to precisely control and vary how much informational influence AI agents and humans should possess. By contrasting human decision certainty to a Bayesian updating model that quantifies the degree of uncertainty in a given situation, we are able to separate informational and normative influence. Specifically, in our study, we operationalise informational influence as adjustment driven by the perceived accuracy of the information, and normative influence as the extra weight assigned to an advice source even when two sources are equally valid and without overt social pressure cues^9,33^. Secondly, the use of an abstract representation of the advisors, where there is no interaction with actual human or AI collaborators, allows us to minimise anthropomorphism and create a level playing field for potential normative influence across the two types of advisors. Thirdly, the information cascade paradigm allows us to quantify the informational and normative influences by estimating how much weight individuals give to specific information sources. Finally, healthcare is a domain in which AI decision aids are becoming widely applied, and therefore this medical decision making scenario offers some timely ecological validity.

### Hypotheses

Theories of epistemic authority suggest that trust in an advisor is functionally determined by their perceived competence and source validity rather than social category^34^. In the context of AI, this aligns with the framework of cognitive trust, where users evaluate agents primarily based on their performance track record and technical reliability^35^. Previous research supports this view, indicating that informational influence is a primary driver of conformity when people face both humans^22^ and non-human agents embodied in computers or robots^16,17,30^. Consequently, from a rational choice perspective, decision makers motivated by accuracy should integrate information independent of its source^22^. Based on this theoretical grounding, we propose an equivalence hypothesis^36^: when informational validity is held constant, AI agents will exert informational influence comparable to human advisors. Thus, our Hypothesis 1 posits that if we introduce AI agents into decision making teams, they will have informational influence similar to the human sources. If this hypothesis is upheld, participants will choose the option supported by more evidence, regardless of whether the advice originates from human or AI advisors.

Previous research suggests that normative influence is evoked more strongly by humans than by non-human agents^16,17,30^ but the degree of normative influence has been confounded by the degree of anthropomorphism in the representation of non-human agents. To distinguish between the two, we minimised and controlled for differences in anthropomorphism presented by human and AI agents using abstract representations. Thus, we propose Hypothesis 2: if we introduce AI agents into decision making teams, they will have less normative influence than the human sources of social conformity in the team. If this hypothesis is upheld, participants should more often choose the diagnosis consistent with their private information (patient symptoms) when human advisors (rather than AI advisors) corroborate the private information. They should also be more confident in choosing this diagnosis when human advisors corroborate the private information.

### Method

#### Participants

We recruited sixty participants online through Amazon Mechanical Turk. This sample size should be sufficient for us to detect moderate to strong effect size in the drivers of social conformity, following previous research^22^ that found an effect size of Cohen’s $d = 0.56$. All participants were from the US, aged over 18, fluent in English, and did not have a background in computer science. We excluded computer scientists because they may perceive AI differently from lay people. To exclude participants with a computer science background, we used a consent form on MTurk asking if they had such a background. Those who answered affirmatively were automatically excluded. We did not distinguish between students and professionals. We did not exclude medical experts as the task was designed for lay people and did not require specialist medical knowledge. Participants made decisions based on probabilistic information and advice from others (human or AI), so we assumed medical professionals would interpret the information similarly to lay participants. Participants received a base rate payment of $\pounds$2 and an additional performance contingent payment. Performance contingent payment is a standard procedure in economic experiments to increase motivation and a similar payment scheme has previously been applied in this medical decision task^22^. Specifically, our payment scheme incentivised accurate answers and well calibrated confidence ratings. Participants received an incentive of 2 pence for each correct decision. If participants’ confidence rating lay within the range of +/− 5% of the correct probability, participants received an additional 3 pence for each trial. Participants earned an average bonus of $\pounds$0.88 per person ($SD = 0.20$) and completed the study in 17.44 minutes ($SD = 8.19$). This payment scheme translates to an hourly wage of $\pounds$9.90 per hour (above the national minimum wage at the time of the study). All experimental protocols were approved by the Psychology Research Ethics Committee at the University of Bath with the reference number: 21-085. All methods were carried out in accordance with relevant guidelines and regulations. Informed consent was obtained from all participants.

#### Design

We adopted a within participants design and counterbalanced the order of conditions. Three independent variables were manipulated: the number of advisors (1–3), if the advisors were human or AI, and towards which disease their advice pointed (appendicitis or sigmoid diverticulitis). For example, if participants’ private information pointed in one scenario towards appendicitis, the private information pointed in another scenario towards sigmoid diverticulitis. These combinations resulted in 52 scenarios with four different posterior probabilities: 0.5, 0.67, 0.80, and 0.89. (All scenarios are listed in the Section A of the Supplementary Material.) All participants completed the 52 scenarios in a randomised order.

Our design allowed us to investigate scenarios with various proportions of humans and AIs; for instance, decisions in which a human provided advice contradicting the opinion of one or two AI advisors. Such contrasts enable us to estimate how much more weight individuals put on AI or human advice, independent of the evidence that AI or human advisors provide in total for a given disease, making it possible to distinguish between normative and informational influence at the individual level.

#### Procedure

At the start of the experiment, participants were instructed to play the role of an assistant physician in a medical decision making task. They had to diagnose the disease of a fictitious patient suffering from either sigmoid diverticulitis or appendicitis. Participants received private information, namely the symptom that the patient was experiencing (vomiting or pain in the abdomen), which was made available to the participant to make their own independent judgement. Participants were informed that the accuracy with which each symptom predicted the disease was 66.7%. Specifically, vomiting predicted appendicitis with a probability of 66.7% (and sigmoid diverticulitis with a probability of 33.3%), while pain in the abdomen predicted sigmoid diverticulitis with a probability of 66.7% (and appendicitis with a probability of 33.3%). To inform each decision, participants were also provided with 1, 2, or 3 existing diagnoses of the patient’s illness, referred to as public information. Participants were told that these diagnoses had come either from human or AI clinicians, and that each clinician had received independent information about the patient, which allowed them to correctly diagnose the patient with a probability of 66.7%. After participants made their decision, they were asked how likely they thought their decision was correct on a scale from 50 to 100%, providing a measure of confidence in their decision, referred to subsequently as *choice confidence*. To minimise the potential for order effects and control for decision fatigue, the order of the scenarios was randomised for each participant.

When the experimental trials started, participants saw a fictitious medical record and received the following instruction: ‘The medical record shows the following information: You are an assistant physician in the internal medicine department of a hospital. At the beginning of your breakfast service, a nurse talks to you. She tells you that a patient who has been brought to hospital yesterday wants to talk to a doctor. You get the patient record below: an AI clinician’s (or several AI clinicians’) diagnosis, and a human clinician’s (or several human clinicians’) diagnosis. The nurse tells you the patient’s symptom. You are asked for a diagnosis and the choice confidence.’

**Fig. 1:**
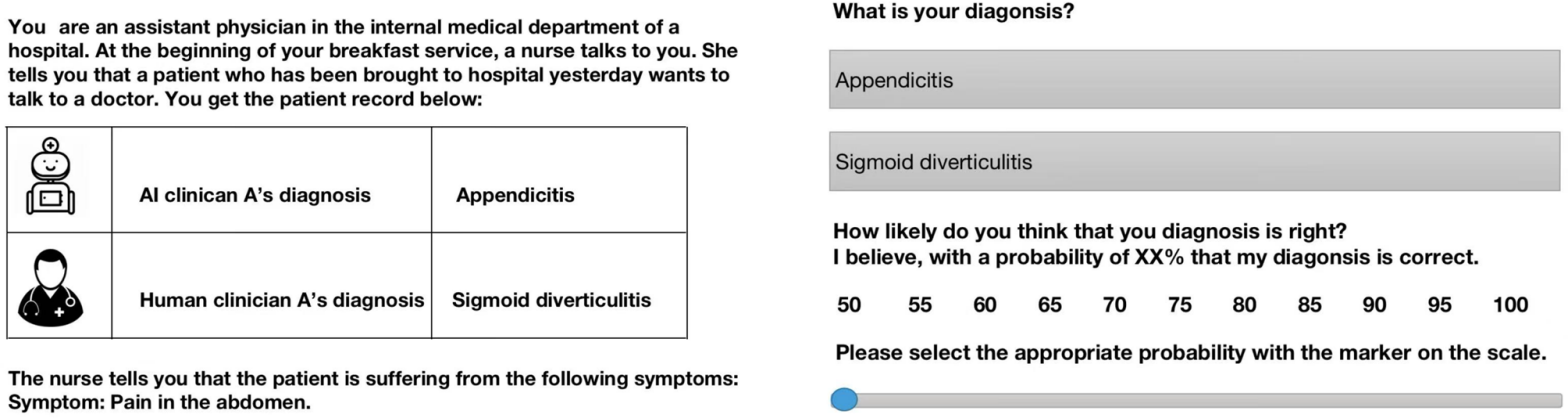
An example decision making scenario presenting (on the left) previous diagnoses from one human and one AI clinician and the symptom abdominal pain. On the right, the participant is to record their diagnosis and confidence level.

Figure 1 shows one scenario as an example. In this scenario, the medical record displays the previous diagnoses (the public information) from just two advisors: the AI clinician diagnoses appendicitis (with an accuracy of 66.7%) and the human clinician diagnoses sigmoid diverticulitis (with an accuracy of 66.7%). The nurse reports pain in the abdomen as the patient’s symptom (the private information) which points with 66.7% probability to sigmoid diverticulitis and 33.3% to appendicitis. In this example, Bayesian updating (formula 1) would predict with 66.7% probability that the patient suffers from sigmoid diverticulitis (the posterior probability). Consequently, if participants simply rationally integrate public information with their private information, the participant should choose the disease sigmoid diverticulitis and state that his diagnosis is correct with a probability of 67%.

### Results

We focused on the following dependent variables in the data analysis: choice percentages indicating how likely participants were to choose the more probable disease (to assess informational influence) and choice percentages indicating how likely participants were to choose the private information (to assess normative influence). In addition, choice confidence provides us in both cases with a measure of how confident participants were. All analyses accounted for the within-group design of the study.

#### Informational influence

If participants use all the evidence provided and are influenced by both private information (symptoms) and public information (advice from others), they should choose the more likely disease more often when more evidence points to it. Thus, participants should choose the more likely disease more often with increasing posterior probability. Similarly, participants should also be more confident in their choice when the posterior probability is higher. The following example shows how we calculated choice confidence for the more probable disease from participants’ choices and their confidence rating: assume that a participant chose appendicitis with a confidence rating of $60\%$. If appendicitis was indeed the likelier disease in this trial, their choice confidence for this option is also $60\%$. If the more probable disease in this trial was, however, sigmoid diverticulitis, the choice confidence for the more probable disease can be calculated as $100\% - 60\% = 40\%$. We aggregated data at the individual level and calculated the average proportion of choices and choice confidence for each level of posterior probability.

**Fig. 2:**
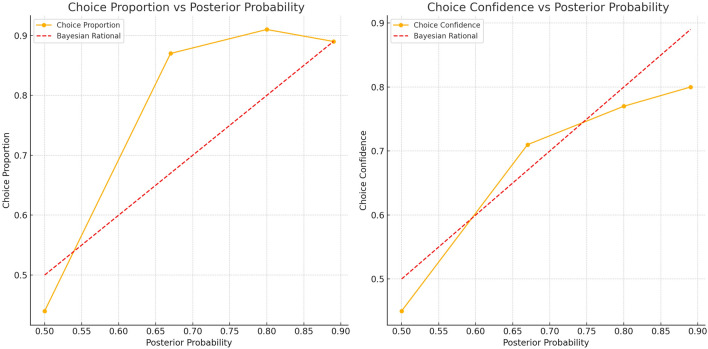
Average Percentage of Choices and Choice Confidence Increased with the Increasing Posterior Probability. The dashed line shows the choice pattern derived from a Bayesian computational reference.

As expected, we found a significant tendency for participants’ average choices and choice confidence to increase with the rising posterior probability, shown in Fig. 2. This trend aligns with the benchmark of a Bayesian rational decision maker, suggesting that participants exhibited certain characteristics of rational decision making within the experimental context. Notably, when the posterior probability was high, the choice proportion approached the ideal pattern predicted by Bayesian theory. This indicates that participants were able to utilise the available information to make reasonable decisions to some extent, and their choices tended towards rationality in situations with clearer information. Specifically, the average choice for the most likely diagnosis increased from 44% ($SD = 0.33$) at a posterior probability of 0.5 to 90% ($SD = 0.20$) at a posterior probability of 0.89. (An *SD* = 33% on the percentage point scale is equal to *SD* = 0.33. All subsequent reports of standard deviation follow the same meaning.) Similarly, choice confidence increased from 45% ($SD = 0.19$) at a posterior probability of 0.5 to 80% ($SD = 0.17$) at a posterior probability of 0.89.

**Table 1:** Proportions and confidence of choices in line with private information at each level of posterior probability (Hierarchical Analysis).

PostPro	0.50		0.67		0.80		0.89	
PI. Fav	H	AI	H	AI	H	AI	H	AI
Scenarios	H:A	AI:A	H:A	AI:A	2H:A	2AI:A	2H:A	2AI:A
	2AI:S	2H:S	AI:S	H:S	AI:S	H:S	PI:A	PI:A
	PI:A;	PI:A;	PI:A;	PI:A;	PI:A;	PI:A;		
	H:S	AI:S	H:S	AI:S	2H:S	2AI:S	2H:S	2AI:S
	2AI:A	2H:A	AI:A	H:A	AI:A	H:S	PI:S	PI:S
	PI:S	PI:S	PI:S	PI:S	PI:S	PI:S		
Choice Prop	69%	57%	87%	81%	91%	85%	89%	89%
GEE Model	$b=0.51$		$b=0.43$		$b=0.56$		$b=0.00$	
(Choice)	$SE=0.22$		$SE=0.29$		$SE=0.26$		$SE=0.40$	
	$p=.023$		$p=.14$		$p=.033$		$p=1.00$	
Choice Con	59%	54%	71%	67%	77%	71%	80%	81%
LMM Model	$b=0.05$		$b=0.04$		$b=0.06$		$b=-0.01$	
(Confidence)	$SE=0.03$		$SE=0.03$		$SE=0.02$		$SE=0.02$	
	$p=.065$		$p=.14$		$p=.009$		$p=.66$	

Choice Prop and Choice Con represent the mean values for each condition. Analysis adjusted for hierarchical data structure. Choice analyzed using Generalized Estimating Equations (GEE); Confidence analyzed using Linear Mixed Models (LMM). *b*: Unstandardized regression coefficient (Human *vs* AI). PostPro: Posterior Probability

**Fig. 3:**
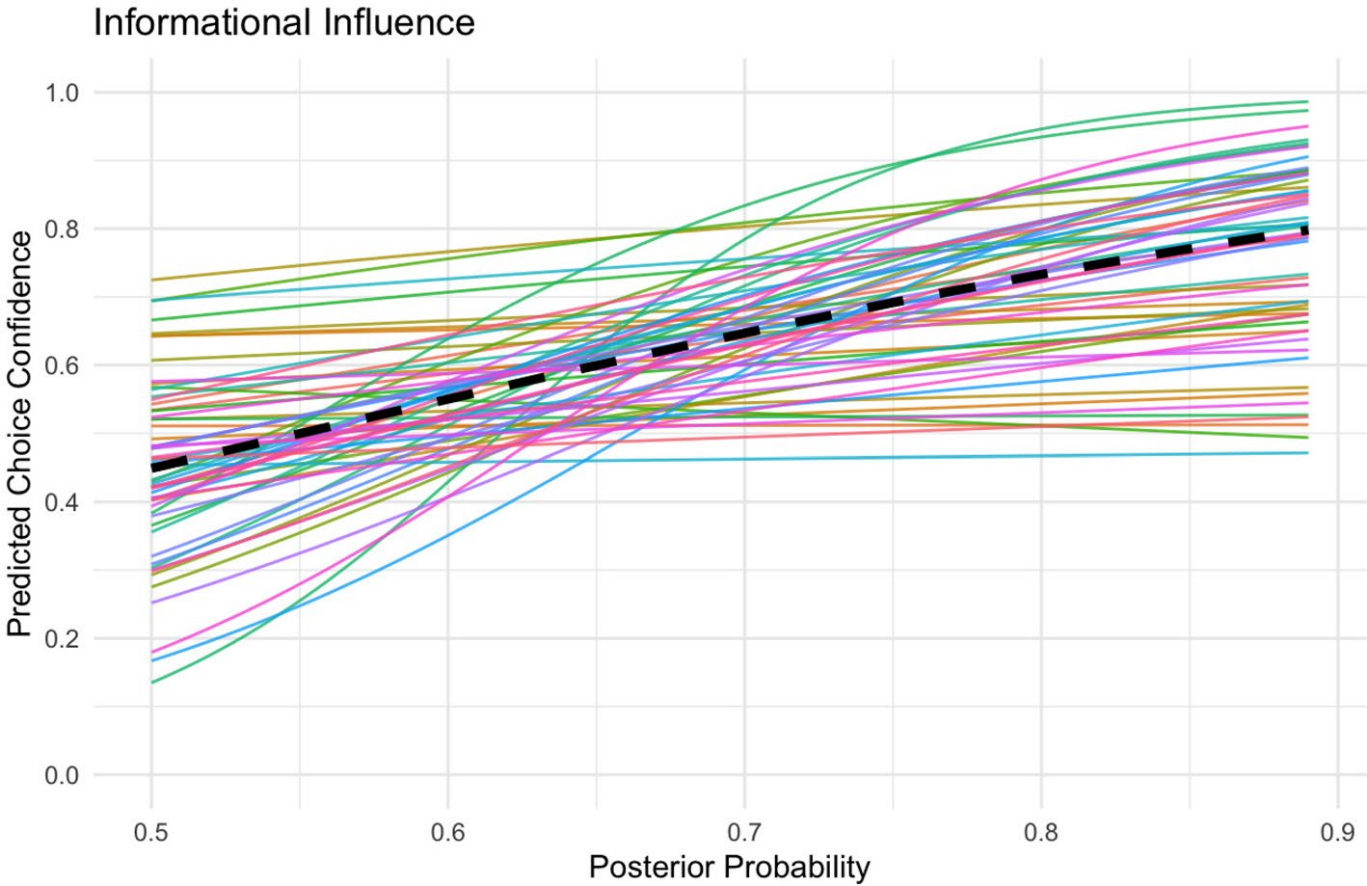
Informational Influence. Participants’ choice confidence as a function of posterior probability for the more likely diagnosis. The black line represents the fixed effect and the light grey lines represent the random effects which are individual slopes.

Using IBM SPSS version 28, we conducted a mixed linear model analysis with the beta family to predict choice confidence with posterior probability (Fig. 3). The model included both fixed effects and random effects. Fixed effects were used to estimate the overall influence of posterior probability on choice confidence, and the effect of the type of advisor (human *vs* AI) on decision confidence. Random effects included a random slope and intercept for each participant, allowing for individual differences in how strongly choice confidence increases with posterior probability, as well as differences in participants’ overall levels of confidence in the chosen diagnosis. This mixed model approach is particularly suited for our within participants design, as it accounts for the correlated data points from each participant across multiple conditions. The results demonstrate a positive relationship between posterior probability and choice confidence, $b = 4.05, SE = 0.47, z = 8.68, p <.001$. This indicates that individuals became on average more confident in their choice the higher the posterior probability is. The variance in slopes across participants shows that for some participants choice confidence increased more strongly with posterior probability than for other participants, $Var(\mu _{1j}) = 11.98, p <.001$. The intercept, that is the overall confidence level, also varied across participants, $Var(\mu _{0j}) = 5.10, p <.001$.

To investigate whether informational influence differed between human and AI advisors, we added the proportion of AI advisors (compared to human advisors) and its interaction with posterior probability to the linear mixed model with beta family. There was no interaction between the proportion of AI advisors and the posterior probability, $b = -0.12, SE=0.35, p =.72$. To rigorously interpret this non-significant result, we complemented the conventional NHST with an equivalence test (TOST) embedded in a linear mixed effects model^36,37^ to test if the informational influence between humans and AIs is equivalent. In the absence of an established theoretical bound and with a fixed sample size, we established the SESOI based on established disciplinary conventions for practical significance, employing a two-step justification procedure. First, we referenced standard conventions in psychology to define a “practically meaningful” difference. While Cohen^38^ defines $d=0.5$ as a medium effect, a large scale meta-analysis of social psychology research^39^ indicates that the average published effect size corresponds to $d \approx 0.43$. Effects smaller than this threshold are often considered trivial or indistinguishable from noise in complex behavioural tasks. Aligning with these benchmarks, we set the base theoretical SESOI at $d = 0.425$ (representing a “small to medium” effect). Secondly, detecting interaction effects in linear mixed models inherently requires larger samples than main effects to achieve equivalent power^40^. For a fixed sample size, the standard error of an interaction term is approximately double that of a main effect. To ensure the equivalence test was rigorously powered to detect the defined meaningful effect, we adjusted the SESOI for the interaction term by a factor of 2 ($\sqrt{4}$), resulting in a final SESOI of $d_{interaction} = 0.425 \times 2 = 0.85$. We explicitly note that this threshold is conservative regarding power but lenient regarding equivalence: it is designed to test whether the difference between Human and AI influence is “large and substantial”. Finally, we fitted a linear mixed model predicting participants’ choice confidence to the most likely diagnosis with the interaction between evidence strength and proportion of human advisors. We mapped the standardized SESOI ($d=0.85$) to the raw scale using the model residual standard deviation ($\sigma _\varepsilon \approx 0.24$), yielding raw equivalence bounds of $[-\Delta , \Delta ] = [-0.204, 0.204]$. The results confirmed equivalence. The 90% confidence interval for the interaction coefficient was $[-0.197, 0.068]$, which lies fully within the equivalence bounds $[-0.204, 0.204]$. Consequently, the TOST procedure was significant ($p <.05$), indicating that the difference in informational influence between AI and human advisors is statistically smaller than the established threshold for a meaningful effect. Thus, while we cannot rule out small or medium-sized differences due to sample size limitations, we can confidently reject the hypothesis that there is a large, substantial disparity in how participants weigh information from AI *vs* human advisors.

Overall, the results support the informational influence hypothesis in human-AI collective decision making. People adjust their choices and choice confidence as a function of how much evidence points towards each disease. Further, they adjust their choices and choice confidence for human advisors and AI alike, suggesting that human advisors do not evoke stronger informational influence than AI advisors.

#### Normative influence

But do human advisors elicit stronger *normative* influence than AI advisors? We first examined, across all the scenarios in which human team members’ or AI agents’ advice corroborated participants’ private information (Table 1), if participants more often followed their private information and were more confident in their choice. We excluded trials in which human and AI advisors’ diagnoses contradicted participants’ private information because such trials reveal only whether participants conform to the advisors or rely on their private information. This does not help us distinguish between the normative influences of human and AI advisors. Additionally, we excluded trials in which humans and AI have the same diagnoses. This allows us to more clearly differentiate the normative influence exerted by human advisors from that of AI advisors, reducing potential noise in the data. Next, we examined separate groups of selected scenarios in which the posterior probability was 0.5, 0.67, 0.8 and 0.89. If human advisors evoke stronger normative influence than AI advisors, then participants’ choices should more often follow their private information and they should be more confident in their choice when human advisors corroborate the private information compared to AI advisors (at the same level of posterior probability). Data were analyzed using Generalized Estimating Equations (GEE) for binary choices because of its robustness in estimating population-averaged effects while correcting for within-subject correlations using robust standard errors, and Linear Mixed Models (LMM) for confidence ratings, which allowed us to explicitly model individual heterogeneity through random intercepts.

Across all scenarios where advisors corroborated private information, participants followed the private information significantly more often when supported by human advisors ($M = 84\%, SD = 0.37$) than by AI advisors ($M = 78\%, SD = 0.41$). A GEE analysis confirmed this main effect of advisor type ($b = 0.38, SE = 0.13, p =.004$). Similarly, participants were significantly more confident in their judgments when human advisors corroborated their private information ($M = 72\%, SD = 0.26$) compared to AI advisors ($M = 68\%, SD = 0.27$). An LMM analysis further validated this difference ($b = 0.04, SE = 0.02, p =.020$).

The table shows that for levels of posterior probability involving uncertainty (0.5) or moderate certainty (0.80), participants chose more often to follow the advice from human advisors than from AI advisors. This stronger normative influence for human advice was statistically significant for scenarios with posterior probabilities of 0.5 and 0.80, although it was not significant for the intermediate condition of 0.67. In the scenarios where posterior probability was 0.89, we observed equal proportions and similar confidence in choosing the private information when human advisors or AI advisors corroborated the private information. This lack of difference ($p = 1.00$) reflects a ceiling effect, which was confirmed by distributional analysis at the 0.89 posterior probability level. Confidence ratings in both conditions exhibited severe negative skewness ($<-1.8$) and high kurtosis ($>2.5$), with over 50% of responses clustering in the highest decile of the scale (0.90–1.00). These metrics indicate that the measure reached saturation at high informational certainty, statistically limiting the variance required to detect potential differences between advisor types.

In conclusion, we found that participants decided more often in line with their private information and were more confident in their decision when advice from humans aligned with the private information than when advice from AI did–particularly when the evidence was not definitive. Therefore, our results support Hypothesis 2, that in human-AI decision making teams, other humans evoke stronger normative influence than AI agents, except in situations of high informational certainty.

#### Normative influence in a Bayesian social influence model

In order to understand how much stronger normative influence is from humans than from AI agents, we estimated within a social influence model the weights that participants put on three information signals: private information, public information from human advisors, and public information from AI advisors. Specifically, by reformulating Bayes’ theorem (2), we can predict the log odds of the posterior probability for disease A (appendicitis) relative to the posterior probability for disease S (sigmoid diverticulitis) with the log odds of receiving an information signal for disease A compared to disease S. The log odds of receiving an information signal for disease A (compared to disease S) can be separated into information signals from the private information, information signals from the human advisors, and information signals from the AI advisors. *n* represents the total pieces of information from different resources including private information, public information from AI advisors and public information from human advisors:

2 $$\begin{aligned} \begin{aligned} \ln \frac{p(A|n_{PI},n_{H},n_{AI})}{p(S|n_{PI},n_{H},n_{AI})} =&\ \beta _{bias} + \beta _{PI} \ln \frac{p(n_{PI}|A)}{p(n_{PI}|S)}&+ \beta _{H} \sum ^{n_H} \ln \frac{p(n_{H}|A)}{p(n_{H}|S)}&+ \beta _{AI} \sum ^{n_{AI}} \ln \frac{p(n_{AI}|A)}{p(n_{AI}|S)} \end{aligned} \end{aligned}$$

Choice confidence here serves as an indicator of participants’ perceived posterior probability. This reformulation allows us to predict, in a mixed linear regression, choice confidence with the separate information signals. We chose a linear mixed model because it allows us to account for both the fixed effects of the information signals (human advice, AI advice and private information) and the random effects of individual differences on how participants weigh these signals. The model estimates an overall intercept $\beta _{bias}$ which represents participants’ initial preference to choose disease A over S. We also estimate three fixed slopes for the information signals: the weight of information from human advisors $\beta _{H}$, the weight of information from AI advisors $\beta _{AI}$, and the weight of private information $\beta _{PI}$. If participants integrate all information signals in strict accordance with Bayes’ theorem, each signal should obtain a weight of 1. A weight higher than 1 indicates that participants weigh this signal more heavily than the computational baseline, while a weight smaller than 1 indicates a more conservative integration of this signal. The model also estimates a random slope and intercept for each participant, thus assuming how much weight individuals place on each information signal can vary across participants.

**Fig. 4:**
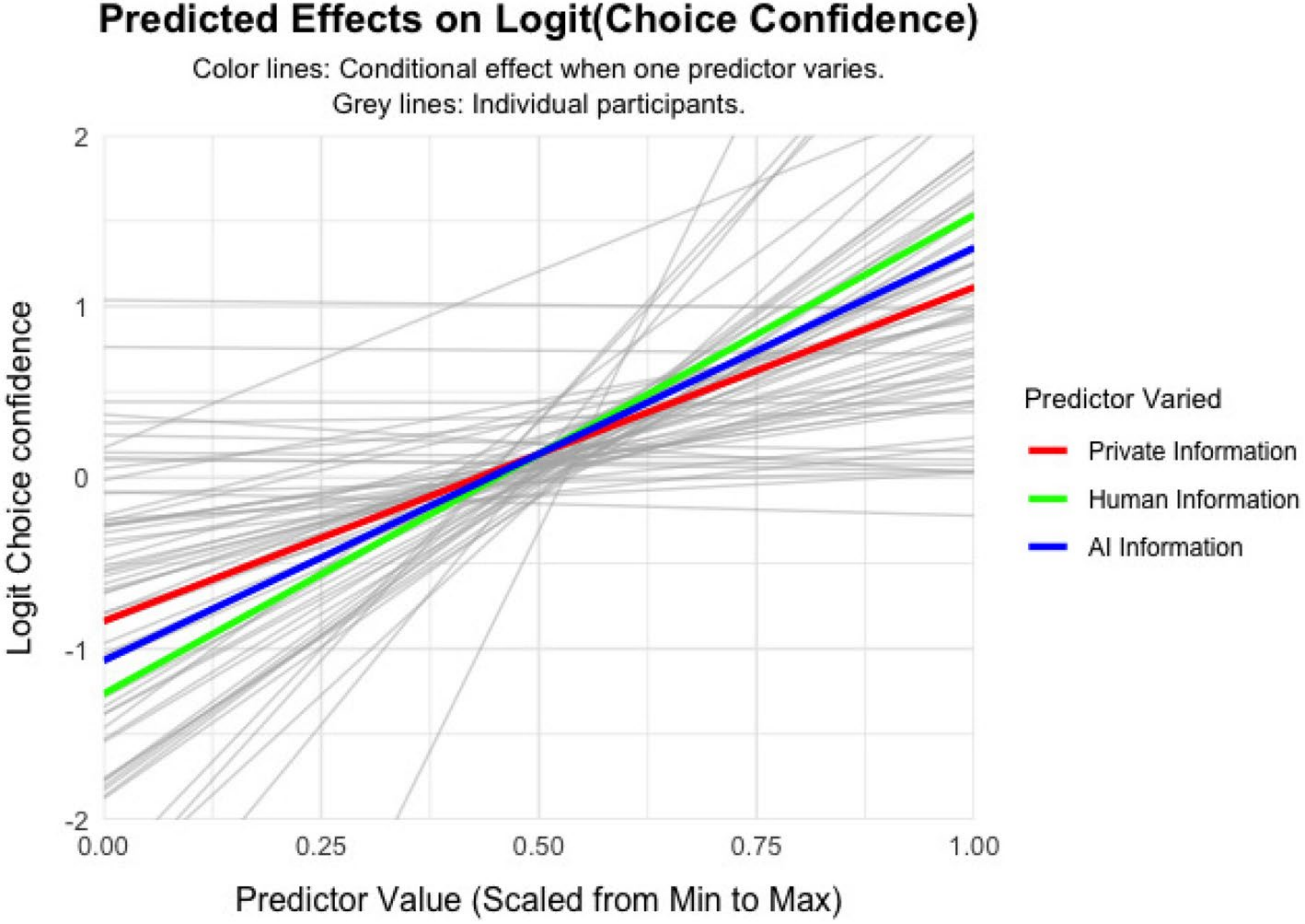
Social Influence Model. Participants’ choice confidence as a function of posterior probability for the more likely diagnosis. Red line: conditional effect of the private information. Green line: conditional effect of human advice. Blue line: conditional effect of AI advice.

The results suggest that participants have a slight preference to choose disease A compared to S, as indicated by a positive intercept $\beta _{\text {bias}} = 0.14$, $SE = 0.05$, 95% CI [0.05, 0.23], $t(59) = 3.0$, $p =.004$. Further, all information weights are above 0, indicating that individuals more confidently choose disease A if the evidence points towards A, $\beta _{PI} = 1.41$, $SE = 0.18$, 95% CI [1.04, 1.77], $t(59) = 7.65$, $p <.001$; $\beta _{H} = 1.00$, $SE = 0.11$, 95% CI [0.78, 1.24], $t(59) = 8.78$, $p <.001$; $\beta _{AI} = 0.87$, $SE = 0.10$, 95% CI [0.67, 1.07], $t(59) = 8.65$, $p <.001$. The slopes varied across participants, indicating that individuals vary in how much weight they put on each information signal, $\textit{Var}(\beta _{PI}) = 1.95$, $SE = 0.37$, $p <.001$; $\textit{Var}(\beta _{H}) = 0.71$, $SE = 0.15$, $p <.001$; $\textit{Var}(\beta _{AI}) = 0.53$, $SE = 0.11$, $p <.001$. The intercept, that is, the preference towards disease A over S, also varied across participants, $\textit{Var}(\beta _{\text {bias}}) = 0.09$, $SE = 0.02$, $p <.001$ (Fig. 4).

To compare the information weights for different resources, we conducted linear hypothesis tests (contrasts) on the fixed effect coefficients derived from the linear mixed model. Results indicated that participants assigned significantly greater weight to their private information compared to advice from human advisors ($\beta _{diff} = 0.40$, $z = 2.04$, $p =.041$) and AI advice ($\beta _{diff} = 0.54$, $z = 2.81$, $p =.005$). This result demonstrates that people valued their own private information more than any advice from others, despite having the same validity. Finally, we found a significant difference between the weights assigned to human advice and AI advice ($\beta _{diff} = 0.14$, $z = 2.69$, $p =.007$). This suggests that human advisors evoked stronger normative influence than AI agents.

#### Discussion of study one

Study one investigated to what extent social conformity influences decision making in human-AI teams and distinguished between two drivers of social conformity: informational and normative influence. We hypothesised that human-AI teams should demonstrate the same degree of informational influence as human teams. Our results revealed that with increasing posterior probability of one alternative, participants more often chose the likelier diagnosis and were also more confident in their decision regardless of whether they received advice from human or AI advisors. This finding supports previous research suggesting that non-human agents may also evoke informational influence^16,17,30^. In addition, going beyond previous studies, our research quantifies this informational influence.

Further, we expected that human-only teams should evoke stronger normative influence than human-AI teams. However, it is important to note that the influence of human advice could be driven by both normative and informational factors. While we hypothesised that the observed effect was primarily normative, one might also consider the possibility that participants perceived human advice as more informationally valid than AI advice, even though both were presented as equally valid. The difference in weighting human versus AI advice suggests that participants may have seen the human advice as more socially acceptable or trustworthy, rather than simply as a more accurate source of information. This aligns with the idea of normative influence, where individuals may conform to human advice because they perceive it as more socially valid, even in the absence of explicit social cues. We found that normative influence is stronger in human only teams than in human-AI teams. When the advice from humans pointed towards the same diagnosis as participants’ private information, participants were more likely to follow their private information and were more confident in their choice than if the advice from AI aligned with participants’ private information. People also put more weight on human advice than on AI advice. Notably, participants weighted the public information from human advisors converged to the theoretical benchmark derived from Bayes’ rule, $\beta _{humans}$ = 1.00, but underweighted the public information from AI advisors, $\beta _{AI}$ = 0.87. This result suggests that people followed the public information from AI less than the public information from humans although both pieces of information had the same validity. It is surprising that Study 1 revealed differences in normative influence between human and AI advisors even in our hypothetical decision making context with limited visual variation. However, research on social perception suggests that humans are remarkably sensitive to even “extremely simple stimuli” and capable of discerning social cues from minimal visual differences^41^. This sensitivity implies that even subtle variations in the description and visual presentation of the advisors, such as avatar styles or interface design, could be sufficient to trigger distinct social perceptions and differential normative influence. Supporting this idea, previous work has demonstrated that changing the description of the advisor modulates its normative influence on decision making even in hypothetical scenarios^22^.

People placed high importance on their private information, $\beta _{PI}$ = 1.41, and overweighted it compared to the public information. This result indicates that individuals in our decision task did not follow blindly the advice of either humans or AI, as research within the Asch paradigm suggests^16,17^, but rather overly followed their own knowledge^42^. As public information from humans is considered appropriately in the decision, this finding opens up the possibility that the reduced normative influence in human-AI teams is a consequence of algorithm aversion, the phenomenon that people follow other humans more than algorithmic advice even when algorithmic advice exceeds human performance^43^. It is worth noting that this algorithm aversion appeared most significant when posterior probabilities are low. This suggests that in situations of high uncertainty, participants place greater weight on human advice than on AI advice. This pattern could become problematic as AI systems improve in reliability, especially in high uncertainty scenarios, potentially leading to suboptimal decisions.

## Study 2: social conformity for human and AI advisors with different levels of credibility

Study 1 demonstrated that social conformity can occur in human-AI teams when all advisors are equally credible, that is they had the same decision accuracy. In real life situations, decision accuracy typically varies between different advisors. For instance, humans may provide more accurate advice for some decisions while in other cases AI advice outperforms any human suggestion. If the number of advisors as well as their accuracy varies, it becomes challenging to determine which advice (human or AI) is more trustworthy. Indeed, people often struggle to interpret probabilities correctly^44^. To facilitate this decision, people may follow simple majority rules^44,45^. They may count the number of (AI and human) advisors speaking for each choice option and choose the option with the highest number of votes, thereby ignoring the accuracy of each advisor. Alternatively, they may trade off variations in decision accuracy against the number of advisors voting for each option. Thus, we ask two research questions: (i) if advisors vary in decision accuracy, will we still observe informational and normative influences in human-AI teams as in Study 1?; and (ii) do individuals rely on majority rules to offset decision accuracy?

We tackled these research questions in a similar research paradigm to Study 1 but varied the decision accuracy of different information sources. This allows us to understand how variations in decision accuracy interact with social influence from several advisors in human-AI teams. Incorporating different decision accuracy also enhances the ecological validity of our study, as real world information sources often vary in accuracy. Additionally, we expanded our social influence model which we applied in Study 1 and include decision accuracy in the model, demonstrating the generalisability of our mathematical model.

### Hypotheses

First, we aim to replicate our findings from Study 1 suggesting that (a) both informational and normative influence affect decisions in human-AI teams and (b) people favour human advisors over AI advisors. Thus, we expect that participants will put more weight on human advisors compared to AI advisors. As a consequence, the AI majority advisors require a greater number than human majority advisors to offset decision accuracy (Hypothesis 1).

Furthermore, it remains an open question how people balance the number of advisors with variations in decision accuracy. When the opinion of the majority of the advisors does not conflict with decision accuracy, we hypothesize that participants will follow the advice of the majority, regardless of whether the majority is human or AI (Hypothesis 2). However, when the opinion of the majority of the advisors conflicts with decision accuracy, we can postulate two competing hypotheses about how majority opinion and decision accuracy interact. Hypothesis 3a: When accuracy conflicts with the majority, people will ignore the accuracy of the advisor and will follow the advice of the majority. Hypothesis 3b: When accuracy conflicts with the majority, people will integrate the decision accuracy with the majority rules, and therefore choose to follow the advice that suggests the most likely diagnosis.

### Methods

#### Participants

Fifty participants (31 men, 19 women, $M_{Age}$ = 30, $SD = 17.04$), participated in an online experiment conducted via Amazon Mechanical Turk. Due to the complexity of the study design, which took a longer time, the payment scheme was higher compared to Study 1. Participants received a base rate payment of $\pounds$2.36. On average, participants completed the study in 28.78 minutes ($SD = 11.06$) and earned an average bonus of $\pounds$2.45 per person ($SD = 0.76$). This payment scheme translates to an hourly wage of $\pounds$9.95 per hour. All experimental protocols were approved by the Social Science Ethics Committee at the University of Bath, with the reference number of 0512-485. All methods were carried out in accordance with relevant guidelines and regulations. Informed consent was obtained from all participants.

#### Design, material, and procedure

The experiment followed the same procedure as in Study 1 except for the following changes. First, we varied decision accuracy between the human and the AI advisors from low (56% accuracy) to high (99% accuracy). All human advisors possessed the same accuracy level in a scenario; all AI advisors possessed the same accuracy level; and the decision accuracy of the AI advisors differed from the human advisors. Further, we manipulated the number of advisors with either the human advisors or the AI advisors being the majority (or minority) in a scenario. We chose 3 advisors as the group size for the majority because a group size of three is sufficient to induce social conformity^46^. We chose 1 advisor as the group size for the minority group. Humans and AI advisors were equally often the majority or minority group.

This design allowed us to examine decisions in scenarios in which decision accuracy agrees with majority rules and in scenarios in which they conflict. In some scenarios, the AI advisors held the majority and had a high decision accuracy, whereas humans were in the minority and had a low decision accuracy. In other scenarios, however, the majority group had a lower decision accuracy than the minority group. Overall, we manipulated four independent variables in a within participants design: the number of advisors (minority and majority), decision accuracy (high and low), types of advisors (human or AI), and towards which disease their advice and participants’ private information pointed (appendicitis or sigmoid diverticulitis). For example, if participants’ private information pointed in one scenario towards appendicitis, the private information pointed in another scenario towards sigmoid diverticulitis. These combinations resulted in 72 scenarios with six different posterior probabilities: 0.56, 0.65, 0.75, 0.81, 0.89 and 0.99 (as listed in section D of the supplementary material). All participants completed these 72 scenarios in a randomized order to minimise the potential for ordering effects.

**Fig. 5:**
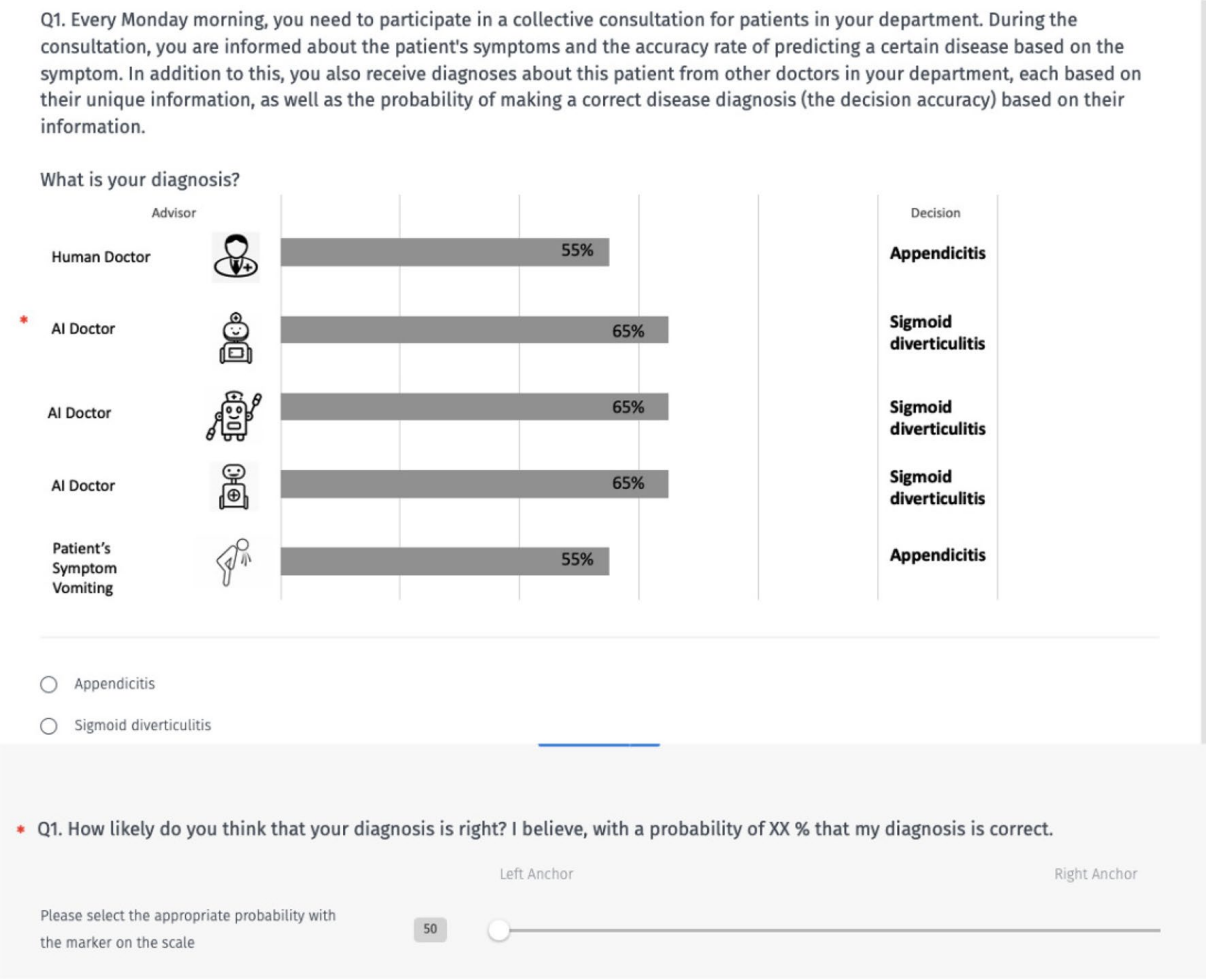
An example decision making scenario in Study 2 illustrating a mixed human-AI team with one human clinician and three AI clinicians. The upper panel shows each advisor’s diagnosis and displayed decision accuracy for each information source, which varied across trials in Study 2 in bar-chart percentages, as well as the patient’s symptom (vomiting). In this example, the three AI clinicians diagnose sigmoid diverticulitis (65% accuracy), while the human clinician and the patient’s symptom support appendicitis (55%). The lower panel shows where the participant records their diagnosis and confidence level.

As the decision accuracy of the advisors varied, we had to display the decision accuracy of each advisor in each scenario. We decided to display the decision accuracy in bar charts with percentages because bar charts are more effective in communicating probabilities than tables^47,48^. Figure 5 shows one scenario as an example. Three AI advisors diagnose sigmoid diverticulitis (with an accuracy of 65%) and one human clinician diagnoses appendicitis (with an accuracy of 55%). The private information (the patient’s symptom) points towards appendicitis, too. In this example, Bayesian updating (Eq. 1) would predict with 81% probability that the patient suffers from sigmoid diverticulitis (the posterior probability). Consequently, if participants simply rationally integrate public information with their private information, the participant should choose the disease sigmoid diverticulitis and state that his diagnosis is correct with a probability of 81%

### Results

#### Informational influence

**Fig. 6:**
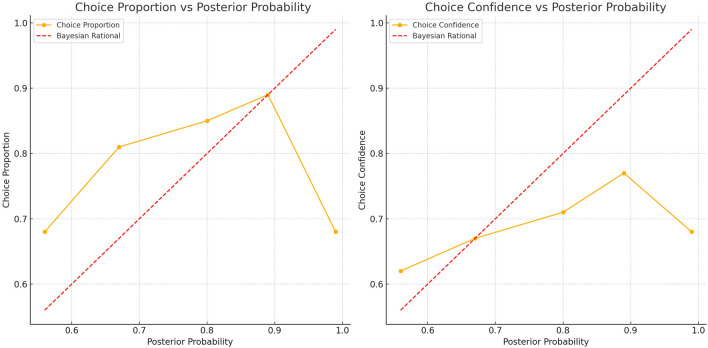
Percentage of Participants’ Choices and Choice Confidence of the most likely choices. The dashed line shows the choice pattern derived from a Bayesian computational reference.

If decision accuracy varies, can informational influence still be observed? If it does, we should find that as the posterior probability for a certain disease increases, participants are more likely to opt for this more probable disease and become more confident in their choice. To investigate this question, we conducted the same analysis as in Study 1. Figure 6 depicts participants’ percentage of choosing the more likely disease and their choice confidence at each level of posterior probability. When the posterior probability was 0.56, the more likely option was chosen in $68\%$ ($SD=0.25$) of scenarios across all participants. Their confidence in these choices was $62\%$ ($SD= 0.18$). The percentage of choosing the more likely option and choice confidence increased with higher levels of posterior probability. Finally, at the highest level of evidence, that is at 0.99 posterior probability, participants selected the most likely choice on average across $68\%$ of scenarios ($SD=0.23$), and their confidence in this choice was $68\%$ ($SD=0.17$). In summary, we observed that participants’ choices and their confidence in selecting the most probable options increased with more evidence for the choice option. Nevertheless, this increase did not perfectly match the proportional growth pattern of a rational Bayesian decision maker, indicating that participants’ decision making processes were influenced by various factors and deviated from strict statistical updating.

**Fig. 7:**
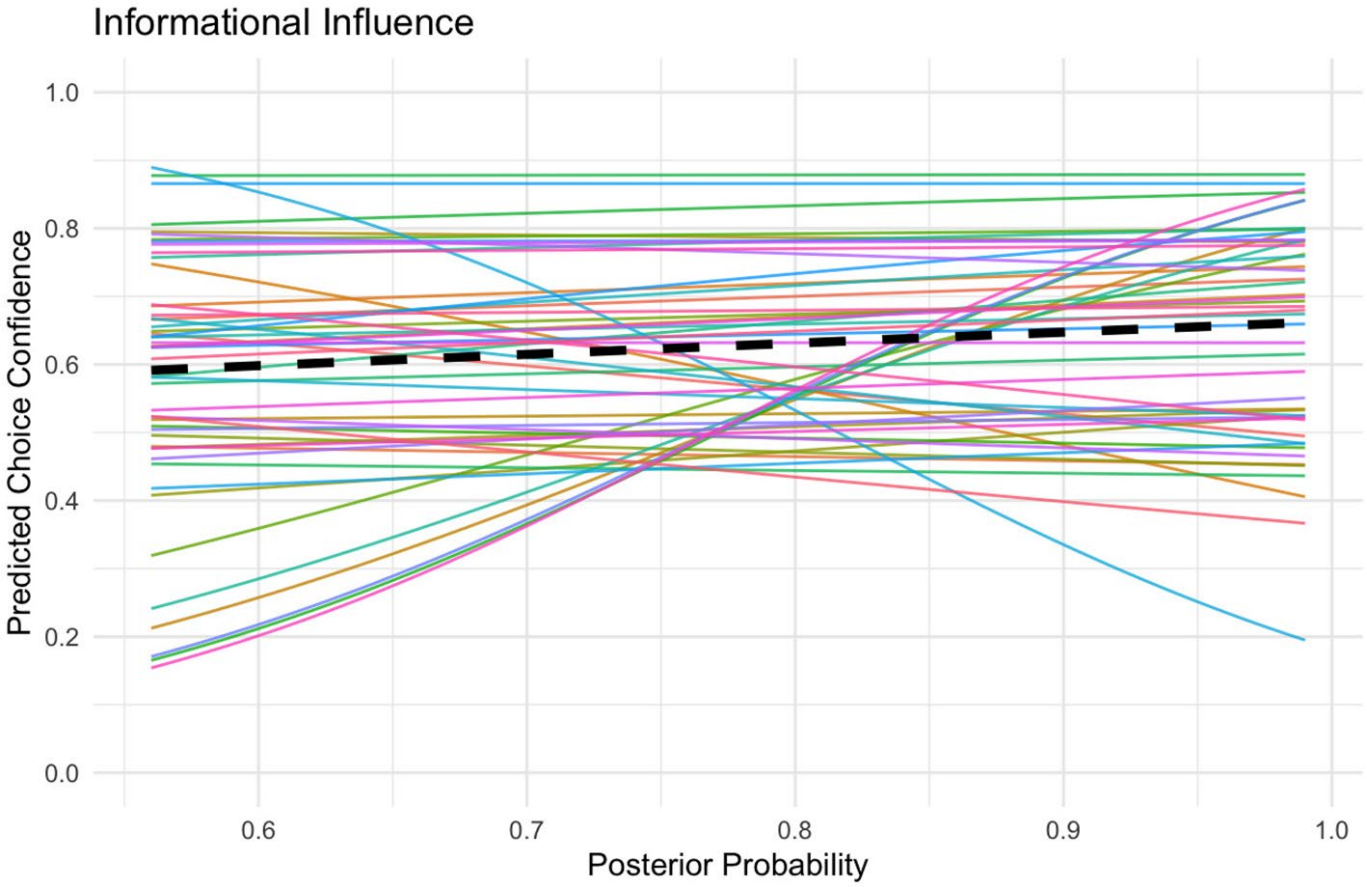
Informational Influence. Participants’ choice confidence as a function of posterior probability for the more likely diagnosis. The black line represents the fixed effect and the light grey lines represent the random effects which are individual slopes.

Using IBM SPSS version 28, we conducted a mixed linear model analysis with beta family to predict choice confidence with posterior probability (Fig. 7) with a random slope and intercept for each participant. We used a mixed linear model because it allows us to account for both the fixed effects (the impact of posterior probability) and random effects (individual differences in decision confidence). The results suggest that individuals become on average slightly more confident in their choice the higher the posterior probability is, $b = 0.70, SE = 0.41, p=0.08$. The variance in slopes across participants shows that for some participants choice confidence increases more strongly with posterior probability than for other participants, $Var(\mu _{1j}) = 7.77, p <.001$. The intercept, that is the overall confidence level, also varied across participants, $Var(\mu _{0j}) = 5.38, p <.001$.

To investigate whether informational influence differed between human and AI advisors, we added the proportion of human advisors (compared to AI advisors) and its interaction with posterior probability to the mixed linear model. There was no interaction between the proportion of human advisors and the posterior probability, $b = -0.17, p =.74$. We also added the proportion of human advisors as a main effect to the mixed linear model, and the results indicated that there was no significant main effect with $b = 0.13, p = 0.75$. To rigorously interpret this non-significant result, we complemented the conventional NHST with an equivalence test (TOST) embedded in a linear mixed effects model^36,37^. We applied the same two-step justification procedure used in Study 1 to define the Smallest Effect Size of Interest (SESOI). We fitted a linear mixed effects model predicting participants’ choice confidence to the most likely diagnosis with the interaction between the posterior probability of the most likely diagnosis and proportion of human advisors as predictors. Using the model’s residual standard deviation ($\sigma _\varepsilon \approx 0.25$), we converted the standardized SESOI into raw equivalence bounds of $[-\Delta , \Delta ] = [-0.212, 0.212]$. The analysis revealed that the interaction coefficient was negligible ($\beta \approx -0.018$, $SE \approx 0.116$). Crucially, the 90% Confidence Interval for the interaction term was $[-0.210,\, 0.173]$, which falls entirely within the equivalence bounds $[-0.212,\, 0.212]$. The TOST procedure was significant ($p <.05$), statistically confirming that the informational influence exerted by human and AI advisors is equivalent. The conventional NHST result for the interaction was non-significant ($p >.05$).

Overall, our findings indicate that while participants showed a tendency to adjust their choice confidence based on the increase in posterior probability, this change in confidence was less pronounced compared to Study 1. This result may indicate that variations in decision accuracy make it harder to estimate how much evidence each option gathers, thus reducing information influence.

#### Normative influence

Does the advice from human advisors exert a stronger normative influence on participants than the advice from AI advisors? Our Hypothesis 1 tested whether participants would assign more importance to the advice of human majority advisors compared to AI majority advisors, similar to our first study. The results indicated no significant differences in the preference for advice from human versus AI advisors.

We conducted an ANOVA analysis to test this hypothesis. This analysis served as a preliminary assessment of advisor type effects on participants’ conformity behavior at the aggregate level. In this ANOVA analysis, we considered advisor type as an independent variable and participants’ choices and choice confidence regarding the majority opinion as dependent variables. If our Hypothesis 1 holds, we expected to observe a main effect of the advisor type on participants’ inclination to select the majority choices and their choice confidence. However, our analysis indicated that there was no significant main effect of the advisor type on the majority party decisions, $F(1, 68) = 0.14, p = 0.71$, $\eta ^2 = 0.002$. Similarly, there was no significant main effect of the type of advisor on the confidence of the participants in their choices of the majority options, $F(1, 68) = 0.01, p=0.91$, $\eta ^2 = 0.00$. These findings imply that the type of advisor, human or AI, does not significantly influence the participants’ choices or their confidence in selecting the majority choices. The results therefore imply that there was no stronger normative influence from human advisors than from AI advisors. However, this analysis is limited as it does not distinguish between the importance people place on their private information compared to any advice they were given. For this reason, we modelled people’s choice confidence with a generalised version of the Bayesian Social Influence Model.

#### Normative influence in a Bayesian social influence model

The Bayesian Social Influence Model allowed us to measure more precisely how much weight participants assigned to their private information, advice from human advisors and advice from AI advisors. We generalised the Bayesian Social Influence Model in equation 3 to account for variations in decision accuracy between advisors.

3 $$\begin{aligned} \ln \frac{P(A|I)}{P(S|I)} = \beta _{\text {bias}} + \sum _{i \in \{H, AI, PI\}} \beta _i \left( \left( N_i = A\right) - \left( N_i = S\right) \right) \ln \frac{\text {Acc}_i}{1 - \text {Acc}_i} \end{aligned}$$

In this equation, $\beta _i$ represents the weight that people assign to each type of information *i*: the weight of information from human advisors $\beta _{H}$, the weight of information from AI advisors $\beta _{AI}$, and the weight of private information $\beta _{PI}$. $\beta _{\text {bias}}$ signifies the initial bias participants hold towards disease A or disease S. Compared to equation 2, the number of pieces of information pointing towards disease A versus disease S, $(N_i = A) - (N_i = S)$, are weighted by their accuracy, through the term $\ln \!\left( \frac{\textrm{Acc}_i}{1-\textrm{Acc}_i}\right)$, rather than being treated as equally informative.

**Fig. 8:**
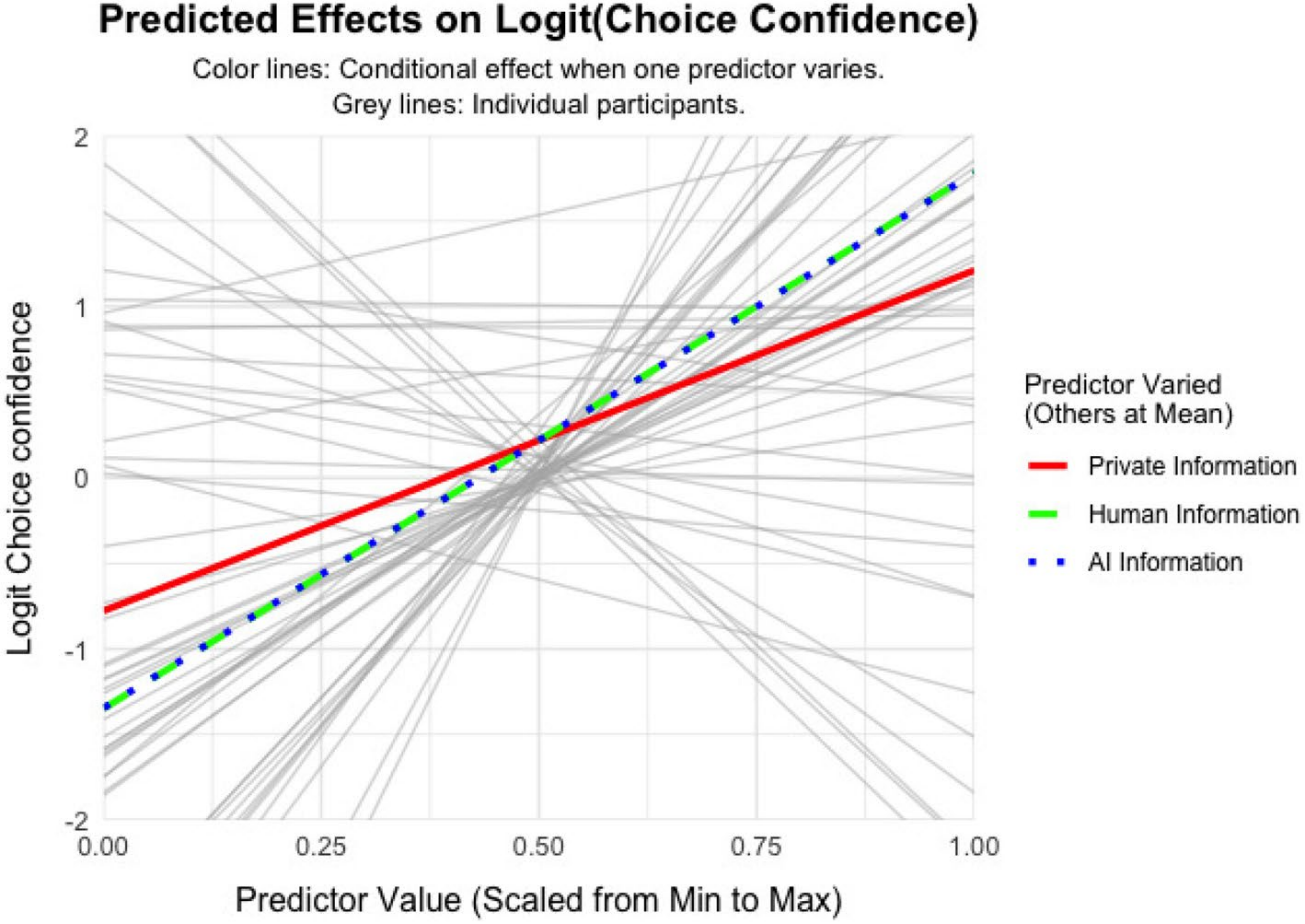
Social Influence Model. Participants’ choice confidence as a function of posterior probability for the more likely diagnosis. Red line: conditional effect of the private information. Green line: conditional effect of human advice. Blue line: conditional effect of AI advice.

The results suggested that participants slightly prefer to choose disease A over S, as indicated by a positive intercept $$\beta _{\text {bias}} = 0.20$$, $$t(49) = 2.61, p <.001, SE = 0.08, 95\% \text { CI } [0.05, 0.38]$$. Further, all information weights are above 0, indicating that individuals more confidently choose disease A if the evidence points towards A, $$\beta _{{PI}} = 0.72,$$ $$t(49) = 4.03,$$$$p < .001,$$$$SE = 0.18,$$$$95\% {\text{ CI }}[0.36,1.08]$$; $$\beta _{{\mathrm{H}}} = 0.302,$$$$t(49) = 6.39,$$$$p < .001,SE = 0.05,$$$$95\% {\text{ CI }}[0.21,0.40]$$; $$\beta _{{AI}} = 0.30,$$$$t(49) = 6.56,$$$$p < .001,SE = 0.05,$$$$95\% {\text{ CI }}[0.21,0.39]$$. The slopes varied across participants, indicating that individuals vary in how much weight they put on each information signal, $$Var(\beta _{{PI}} ) = 1.55,$$$$SE = 0.32,p < .001;$$$$Var(\beta _{H} ) = 0.11,$$$$SE = 0.02,p < .001;$$$$Var(\beta _{{AI}} ) = 0.10,$$$$SE = 0.02,p < .001$$. The intercept, that is the preference towards disease A over S, also varied across participants, $$Var(\beta _{{{\mathrm{bias}}}} ) = 0.33,$$$$SE = 0.07,p < .001$$ (Fig. 8). The findings reveal that the weights assigned to private information, human information and AI information were all below 1. This suggests that participants integrated the available information cautiously and expressed lower choice confidence compared to the theoretical expectations of the Bayesian model.

We compared the information weights using linear hypothesis tests (contrasts) on the fixed effect coefficients derived from the linear mixed model. Results indicated that participants assigned significantly greater weight to their private information compared to advice from human advisors ($\beta _{diff} = 0.42$, $z = 2.61$, $p =.009$) and AI advisors ($\beta _{diff} = 0.42$, $z = 2.65$, $p =.008$). This result demonstrates that people valued their own private information more than any advice from others, despite having the same validity. Finally, we found no significant difference between the weights assigned to human advice and AI advice ($\beta _{diff} < 0.001$, $z = 0.03$, $p =.973$). These findings show that both human and AI advice exerted a smaller influence on choice relative to private information. Further, when the informational context became more complex compared to Study 1, human advice was lo longer preferred over AI advice. This suggests that the presence of explicit and heterogeneous accuracy cues may have prioritised informational weighting over implicit normative considerations, leading to a similar level of influence for both AI and human advisors.

#### Balancing decision accuracy and majority advice

The second research question asked how people balanced the number of advisors with variations in decision accuracy. To investigate this research question, we conducted a unified analysis using mixed effects models, specifying two separate models defined by their respective dependent variables. For the first model, the dependent variable was participants’ choices to follow the majority’s diagnosis. We coded this as a binary outcome: 1 for trials where participants followed the majority advice and 0 for trials where they did not. This variable was analyzed using a Generalized Linear Mixed Model (GLMM) with a binomial link function. For the second model, the dependent variable was participants’ choice confidence in the majority’s diagnosis, which was analyzed using a Linear Mixed Model (LMM).

Both models incorporated the same set of independent variables (fixed effects) to test the experimental hypotheses. The first independent variable was Advisor Type, a categorical variable distinguishing whether the majority consisted of human or AI agents, with the “AI” condition set as the reference level. The second independent variable was Majority Accuracy, a categorical variable capturing the consistency between the majority opinion and the statistically most likely diagnosis. This variable was coded based on the relative validity of the majority opinion compared to the minority. Specifically, the “High Accuracy” condition (reference level) was defined as trials where the majority advisor’s decision accuracy was superior to the minority’s (e.g., Majority 70% *vs* Minority 55%). This condition served as the baseline for testing Hypothesis 2. Conversely, the “Low Accuracy” condition was defined as trials where the majority’s accuracy was inferior to the minority’s (e.g., Majority 55% *vs* Minority 70%), creating a conflict scenario used to adjudicate between Hypothesis 3a and Hypothesis 3b. To account for the repeated measures design and unobserved individual heterogeneity, random intercepts for participants were included in all models.

**Fig. 9:**
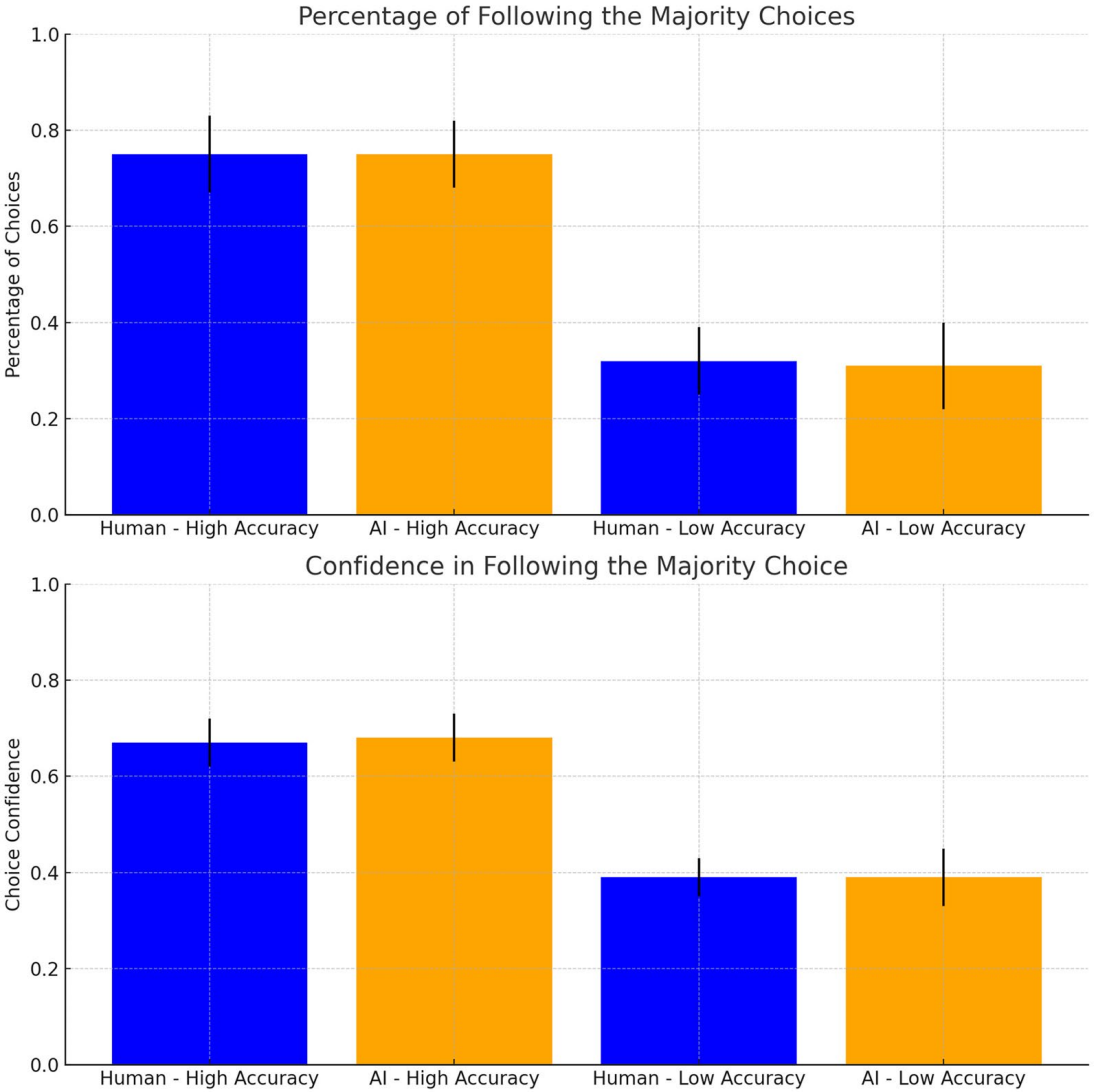
Average choice percentage (upper panel) and choice confidence (lower panel) for following the decision of the majority party. The error bars represent the standard deviation (SD).

First, we tested Hypothesis 2, which postulated that when the opinion of the majority of the advisors does not conflict with decision accuracy, participants will follow the advice of the majority, regardless of whether the majority is human or AI. If this hypothesis holds, we should observe a significant baseline tendency to follow the majority in the high accuracy condition but no significant statistical difference between the human and AI advisor types. The results fully supported these predictions. Descriptively, we found that participants were more likely to follow the majority choice when the majority also had a higher decision accuracy, both for humans ($M =.75$, $SD=0.08$) and for AI ($M =.75$, $SD=0.07$), compared to situations in which the majority had a lower decision accuracy (humans: $M =.32$, $SD=0.07$; AI: $M =.31$, $SD=0.09$), as shown in Fig. 9. The model estimated a significant and high baseline probability of participants’ choices to follow the majority (Choice Intercept: $\beta = 1.49, SE = 0.23, z = 6.54, p <.001$), confirming that majority opinion exerts a strong influence. Crucially, consistent with the hypothesis, this influence was independent of the advisor’s identity: we observed no significant main effect of Advisor Type on participants’ likelihood to follow the majority ($\beta = -0.04, SE = 0.12, z = -0.30, p =.766$). A similar pattern was observed for choice confidence. Participants were more confident in following the majority choice if the majority had high accuracy (human: $M =.67, SD=0.05$; AI: $M =.68, SD=0.05$) compared to low accuracy (human: $M =.39, SD=0.04$; AI: $M =.39, SD=0.06$) as shown in Fig. 9. While the baseline confidence was high, participants’ choice confidence in the majority’s diagnosis did not differ significantly between Human and AI advisors (Confidence: $\beta = -0.003, SE = 0.01, t = -0.26, p =.793$). These findings confirm that participants are equally willing to follow a majority consensus whether it originates from humans or AIs.

Next, we examined how majority influence is affected by decision accuracy. Our goal was to distinguish between two possibilities: ***Hypothesis 3a*** (blind following), which suggests participants will follow the majority regardless of information accuracy, and ***Hypothesis 3b*** (integration of accuracy), which suggests participants will penalise the majority when it is wrong. If Hypothesis 3a were correct, we would expect the main effect of Majority Accuracy to be non-significant. Conversely, if Hypothesis 3b holds, we should observe a significant negative effect of Majority Accuracy, reflecting a sharp decline in participants’ choices to follow the majority when it conflicts with the correct diagnosis. The results provided strong support for Hypothesis 3b. The GLMM model revealed that the accuracy of the majority had a major impact on participants’ choices. Specifically, while participants frequently followed the majority when it was correct, their likelihood of doing so dropped drastically when the majority opinion conflicted with the most likely diagnosis. The statistical estimate confirmed this sharp decline (Low Accuracy condition: $\beta = -2.31$, indicating a strong negative effect; $SE = 0.12, z = -19.15, p <.001$). In simpler terms, when the majority was wrong, participants were significantly less likely to follow the majority’s diagnosis. This pattern was mirrored in the participants’ choice confidence. As expected under Hypothesis 3b, participants’ choice confidence in the majority’s diagnosis decreased significantly in these conflict scenarios compared to when the majority was accurate ($\beta = -0.29$, indicating a drop in confidence; $SE = 0.01, t = -23.18, p <.001$).

Finally, we examined whether this integration of decision accuracy and majority behaviour depended on whether the advisors were human or AI. If the integration of decision accuracy and majority opinions is a general strategy, we should observe no significant interaction between Advisor Type and Majority Accuracy. The statistical analysis supported this prediction, revealing no significant difference between the two types of advisors. Specifically, regarding the participants’ choices to follow the majority, the interaction term yielded a regression coefficient of $\beta = -0.01$ ($SE = 0.17$, $z = -0.08$, $p = 0.937$). A similar pattern was observed for choice confidence, where the interaction term showed a coefficient of $\beta = 0.004$ ($SE = 0.02$, $t = 0.21$, $p = 0.831$).

### Discussion

Study 2 had two goals. First, we aimed to generalise results from Study 1 to advisors with variations in decision accuracy and investigate to what degree we still observe informational and normative influences in human-AI teams. Replicating our findings in Study 1, we find evidence for informational influence: more evidence for the most likely choice increased both the probability that participants chose this option and their choice confidence, but results were less strong and coherent compared to Study 1. To investigate the impact of normative influence, we generalized the Bayesian Social Influence Model to account for variations in decision accuracy. Replicating Study 1, we found that participants put higher weight on their private information than on human or AI advice, suggesting again only a minor role of normative influence. However, participants integrated all information sources conservatively relative to the theoretical baseline. We observed neither a stronger informational nor a stronger normative influence of human advisors compared to AI advisors. These results suggest that individuals may prioritise informational cues over social identity once variations in advisor accuracy are explicitly introduced, potentially attenuating the normative advantage for human peers observed in simpler contexts.

Secondly, we aimed to understand how individuals balance trade-offs between decision accuracy and majority influence and if this trade-off differs between AI and human advisors. We found that individuals were more willing to follow the majority decision when decision accuracy of the majority advisors was high and consistent with the majority advice. However, contrary to our expectation that advice from a human majority would be weighted more heavily than advice from an AI majority, willingness to follow the majority advice did not depend upon the type of the advisor or its interaction with decision accuracy. Finally, we directly tested if individuals utilised majority rules, ignoring decision accuracy, or integrated majority advice with decision accuracy. Our findings reveal that participants tend not to follow the majority opinion when there is a conflict between majority rules and decision accuracy. However, this does not imply that they entirely ignore majority rules. In fact, our data analysis indicates that when these two criteria align, participants are more likely to adhere to the majority opinion. However, when the two criteria conflict with each other, participants are more likely to adhere to the most likely diagnosis. It is worth noting that further analysis indicates that decision accuracy plays a stronger role than the majority rules. Thus, rather than using one strategy, majority rules or decision accuracy, to offset the other, individuals integrate the majority opinion with decision accuracy to guide their judgments.

## General discussion

Our research aimed to distinguish between two drivers of social conformity in decision making within human-AI teams: informative and normative social influence. Our work adds to this literature by investigating to what extent individuals conform to multiple, potentially contradictory pieces of advice in mixed human-AI teams, thereby differentiating between informational and normative influence within a rigorous Bayesian Social Influence Model. We found that humans and AI elicited informational influence to the same degree (Study 1 and 2), albeit decision makers integrated advice from both types of advisor more conservatively when decision accuracy varied (Study 2). These findings highlight that individuals’ social conformity behavior is often driven by the desire to gather information and optimise decision accuracy rather than blindly following the majority.

Results on normative influence were mixed. In both studies, individuals put more weight on their private information than on human or AI advice; human advice influenced their decisions more than AI advice when advisors were equally reliable (Study 1) but not when decision accuracy varied between advisors (Study 2). This discrepancy suggests that the preference for human advice is likely context dependent. In particular, it may be more likely to emerge when advisors are presented as equally reliable (Study 1) and to weaken when explicit source-specific accuracy cues are available (Study 2).

While we did not explicitly manipulate cognitive mechanisms, we suggest three theoretical interpretations to explain these discrepancies. First, as a speculative explanation consistent with a cognitive resource account, we hypothesise that the need to consider variations in decision accuracy may impose an additional cognitive load on the decision maker^49^. As a result, participants may fail to update their knowledge appropriately and underweight all information they receive, as suggested for informational influence^15^. The need to cope with this increased load may also affect people’s preferences for human compared to AI advice.

Without differences in decision accuracy (Study 1), we found that normative influence had a greater impact on human teams compared to human-AI teams. This finding could potentially be interpreted through the lens of algorithm aversion (the preference for human advice over AI advice)^50^, suggesting a possible baseline preference for human advice when advisors are presented as equally reliable and explicit source-specific accuracy cues are absent. However, the disappearance of this effect in Study 2 implies that this aversion is not a stable, immutable bias. Instead, it appears to be a context dependent social heuristic that operates primarily when information is ambiguous. We propose cue salience as a potential interpretation: in Study 2, the requirement to track varying accuracy levels likely made informational cues (competence) more salient than social cues (source identity). Consequently, the cognitive effort directed toward processing validity may have suppressed the normative default of favouring humans, leading to a convergence of influence where informational utility surpassed normative preference. To quantify these dynamics, we relied upon the Bayesian Social Influence Model as our primary analytical framework. While Bayesian updating often serves as a good descriptive model of the reasoner in social contexts^51^, it functions here as a computational benchmark. It remains possible that other descriptive models could provide alternative accounts of the underlying cognitive mechanics, though the Bayesian framework offers a precise metric for identifying systematic deviations.

An alternative, though untested, interpretation is memory based. In Study 1, information accuracy was presented only once, which may have increased participants’ reliance on simpler heuristics (e.g., source type) during later judgements, particularly when accuracy information was not visually reinforced during the decision phase. By contrast, in Study 2, the requirement to track accuracy levels per trial may have focused attention on the informational content, reducing the reliance on source heuristics. While we offer these cognitive load, cue salience and memory-based interpretations as plausible explanations for our behavioural findings, our current experimental design does not directly test them. Future research is needed to confirm these cognitive mechanisms.

Our second study further indicates that when individuals were presented with majority rule and probabilistic decision accuracy simultaneously, they did not rely solely on one strategy. Instead, they integrated both strategies to inform their decision making. Our data analysis reveals that participants are more likely to follow the majority opinion when the two criteria align. Conversely, when the criteria conflict, participants tend to follow the most likely diagnosis. This suggests that individuals do not rely solely on either majority rules or decision accuracy; instead, they integrate both to inform their judgments. If decision accuracy were the sole focus, the presence of a majority would be irrelevant when the criteria align. Similarly, if the majority opinion were the only consideration, the accuracy of the decision would not affect adherence to the majority when the criteria conflict.

## Contribution and practical applications

Our research provides valuable contributions across multiple disciplines, including human-computer interaction (HCI), social psychology, behavioural economics and organisational behaviour. From a social psychology perspective, we advance the understanding of informational and normative influence, originally articulated by Deutsch and Gerard^15^, by extending it from human-only teams to human-AI teams. Using the information cascade paradigm and the Bayesian updating model from behavioural economics^22^, we quantify the role these influences play in mixed-team social conformity. Our findings reveal that, while the informational influence is similar in both settings, the normative influence is weaker in the human-AI teams and disappears when accuracy cues are salient.

These insights have substantial practical implications for the HCI community. Our research shows that advice from multiple AI can increase users’ confidence in AI, suggesting that “algorithm aversion”^52,53^ is context dependent and may be mitigated by increasing informational transparency or by presenting forecasts from multiple AI. This consequence of social conformity may be of particular importance whenever individuals undervalue the accuracy of a single AI’s prediction. For example, users may be wary of the forecasts from a single decision tree but may trust its advice more if other machine learning algorithms or AI generate a consistent prediction. Choosing whether to provide advice from one AI, multiple AI, or to aggregate their advice in a single forecast is a design choice a developer who implements AI advice may make. These design choices come with benefits as well as potential risks. Presenting advice from several AI can increase its credibility but also creates opportunity for manipulation. For example, if teams of AI agents provide consistent fake or simply incorrect information, people will struggle to ignore this information and to insist on their own judgments. In practical applications, developers should carefully weigh these risks and benefits, including ethical considerations, and prioritise transparency in design choices. Furthermore, consistent with previous research^54,55^, our findings suggest that people may struggle to appropriately weigh advice when advisors differ in decision accuracy. This has practical relevance because, in real world settings, advisors rarely provide equally valid advice. From a practical standpoint, interface designers may therefore consider state of the art practices in risk communication to present variations in decision accuracy. For instance, expressing risk as “20 out of 100 people” instead of “20%” can help people better grasp the magnitude of the risk^56^. Besides, designers may also consider adapting the interface to the information processing abilities of the user, customising the complexity and display of the information being presented.

From an organisational behaviour perspective, our study highlights distinct perceptions of humans and AI consistent with previous research^57^. While both exhibit similar informational influence, humans may be perceived as better suited for normative-driven tasks, such as issuing commands, conveying organisational culture or offering emotional support. This suggests a strategic division of labour that leverages these complementary strengths, optimising team performance. For example, in healthcare, AI systems could deliver precise diagnoses while human doctors promote patient adherence and provide compassionate care, leading to improved patient outcomes.

## Limitations and future work

The study reported here is limited in the following ways. First, participants in our study were lay people but had to imagine themselves as trained medical experts. In reality, medical experts may perceive AI decision aids differently from the general public. For instance, experts tend to hesitate before using AI advice^58^. We chose lay people as participants because we were interested in how people take advice in uncertain, complex decision scenarios about which they possess little knowledge. It would be interesting to compare how advice takers with varying degrees of prior knowledge, from lay persons to experts, respond to advice from mixed AI-human teams in complex decision scenarios. Secondly, setting our study in a healthcare context may enhance its ecological validity in that domain but it may not generalise to other domains, such as financial or political decision making. As in many experiments, we face the trade-off between external validity and internal validity. In this research, we prioritised internal validity because we sought to disambiguate informational and normative influence. The medical decision making scenario serves here as a model that we hope can be extended to other settings as we continue to investigate social conformity in human-AI teams. The decision context is simple enough to understand for lay persons but the decision situation is still relevant for medical experts. Furthermore, the task can easily be adjusted to accommodate AI and human advisors with different predictive accuracy or even missing accuracy information, as one would expect in real world contexts. Thus, the present research paradigm and results may be adapted to other domains. Future studies should investigate how individuals adopt advice in more ecologically valid conditions, where advice accuracy is less clear. Further, it is possible that AI advisors are perceived as a single entity, whereas human advisors are treated as independent of each other. This could affect how participants weigh the advice from AI and humans. Future work could explore how individuals perceive the independence of AI signals and develop methods to improve this understanding. In addition, we acknowledge that individuals may express their choice confidence differently^59^, where under-confident individuals may be more accurate than over-confident ones. Although we assumed a consistent metric for confidence expression, future research could explore how individual differences in confidence affect decision making. Finally, in real world medical contexts, the hierarchical status of the advisor plays an important role in eliciting social conformity. In our experiments, participants were not informed about the hierarchical structure. Future research should consider these factors when examining human-AI social conformity.

## Conclusion

Decision making in human-AI teams still lacks theoretical integration with research in related fields and a solid empirical basis^60^. Our research shows that AI advisors elicit similar informational influence as human advisors. Normative influence was more pronounced for human advice when the advisors were equally reliable; this effect did not replicate when advisor accuracy varied, suggesting that phenomena like algorithm aversion in mixed teams is likely context dependent rather than universal. Developers should carefully balance benefits and risks in the design of AI-assisted decision environments, avoid presenting advice in ways that may unintentionally encourage inappropriate reliance on human or AI sources, and consider adaptive interfaces to prevent cognitive overload when multiple advisors provide input. Our research suggests the potential value of leveraging the distinct normative influences of humans and AI, particularly when human and AI advisors are presented as equally accurate, to support a more effective division of labour and foster complementary human-AI collaboration.

## Electronic supplementary material

Below is the link to the electronic supplementary material.

Supplementary Material

## Data Availability

All the data collected from the participants in the present study has been uploaded onto the Open Science Framework (OSF). Here is the anonymous link to access our data sets of the study one: Data sets 1 and the study two Data sets 2. We have also pre-registered and made transparency change on OSF for study 2 as it involved more substantive design and analytic refinements compared to study 1. Here is the link to access our pre-registration.
