# Supplementary material for: Drivers and influence of social conformity on decision making in human-AI teams
Source: Sci Rep. 2026 Mar 13;16:13438. doi: 10.1038/s41598-026-43042-5 (PMC13111705; doi:10.1038/s41598-026-43042-5)
Supplement: Supplementary file 1 — Supplementary Material [file 41598_2026_43042_MOESM1_ESM.pdf]

Supplementary Material

A. Overall Scenarios Construction of Study One

Section A lists the 52 scenarios (or trials) constructed for Study One. The column "Public Information" states for each trial if the advice from each single advisor, AI or human (abbreviated with H), points towards disease A (Appendicitis) or S (Sigmoid Diverticulitis). Multiple advisors are separated by a comma. The column "Private Information" states for each trial if the symptom in that trial points towards disease A (Appendicitis) or S (Sigmoid Diverticulitis). The column "Posterior probability" indicates the posterior probability for the most likely disease. The counterbalance design is considered throughout all scenarios to overcome the ordering effect.

Table 1: Scenario Construction of the Study One

Trials	Public Information	Private Information	Posterior Probability
1	H: A	S	0.5 for A or S
2	H: S	A	0.5 for A or S
3	H: A, H: S, H: S	A	0.5 for A or S
4	H: A, H: S, H: A	S	0.5 for A or S
5	AI: A	S	0.5 for A or S
6	AI: S	A	0.5 for A or S
7	AI: A, AI: S, AI: S	A	0.5 for A or S
8	AI: A, AI: S, AI: A	S	0.5 for A or S
9	H: A, AI: S, AI: S	A	0.5 for A or S
10	H: S, AI: A, AI: A	S	0.5 for A or S
11	AI: A, H: S, H: S	A	0.5 for A or S
12	H: A, AI: S, H: A	S	0.5 for A or S
13	AI: A, H: S, AI: S	A	0.5 for A or S
14	AI: S, H: A, AI: A	S	0.5 for A or S
15	H: A, AI: S, H: S	A	0.5 for A or S
16	H: S, AI: A, H: A	S	0.5 for A or S
17	H: A, H: S	A	0.67 for A
18	H: S, H: A	S	0.67 for S
19	H: A, H: A	S	0.67 for A
20	H: S, H: S	A	0.67 for S
21	AI: A, AI: S	A	0.67 for A
22	AI: S, AI: A	S	0.67 for S
23	AI: A, AI: A	S	0.67 for A
24	AI: S, AI: S	A	0.67 for S
25	H: A, AI: S	A	0.67 for A
26	H: S, AI: A	S	0.67 for S
27	H: S, AI: A	A	0.67 for A
28	H: A, AI: S	S	0.67 for S
29	H: A, AI: A	S	0.67 for A
30	H: S, AI: S	A	0.67 for S
31	H: A	A	0.89 for A
32	H: S	S	0.89 for S
33	AI: A	A	0.89 for A
34	AI: S	S	0.89 for S
35	AI: A, AI: S, AI: A	A	0.89 for A
36	AI: S, AI: A, AI: S	S	0.89 for S
37	H: A, H: S, H: A	A	0.89 for A
38	H: S, H: A, H: S	S	0.89 for S
39	H: A, AI: S, AI: A	A	0.89 for A
40	H: S, AI: A, AI: S	S	0.89 for S
41	AI: A, H: S, H: A	A	0.89 for A
42	AI: S, H: A, H: S	S	0.89 for S
43	AI: A, H: S, AI: A	A	0.89 for A
44	H: A, AI: S, AI: S	S	0.89 for S

Continued on next page

Trials	Public Information	Private Information	Posterior Probability
45	H: A, AI: S, H: A	A	0.89 for A
46	AI: A, H: S, H: S	S	0.89 for S
47	H: A, H: A	A	0.89 for A
48	H: S, H: S	S	0.89 for S
49	AI: A, AI: A	A	0.89 for A
50	AI: S, AI: S	S	0.89 for S
51	H: A, AI: A	A	0.89 for A
52	H: S, AI: S	S	0.89 for S

Notes: H: human information, AI: AI information, A: Appendicitis, S: Sigmoid Diverticulitis

B. Participant Instruction of Study One

Dear Participant

This experiment investigates decision-making under uncertainty in the medical domain. Your task is to imagine yourself in 52 situations that may occur in a hospital and to make specific decisions. Imagine yourself in the position of an assistant physician. Your job is to decide whether, in the situations described, a patient is suffering from either sigmoid diverticulitis or appendicitis. It is not possible for a patient suffers from both diseases simultaneously. Both diseases are equally likely if you do not receive any further information: With 50% probability, the patient is suffering from sigmoid diverticulitis, and with 50% probability, he is suffering from appendicitis. Both diseases manifest themselves mostly through the same symptoms (mild fever, weakness, abdominal pain). In each of the situations described below, you will, however, receive further information about symptoms that speak for one or the other disease and also be able to read the diagnoses of other clinicians. The following symptoms speak rather for the existence of one of the two diseases. Vomiting: The symptom' symptom' vomiting" speaks for appendicitis because: with the disease appendicitis, the occurrence of the symptom "vomiting" is more likely than with the disease sigmoid diverticulitis. With the disease appendicitis, this symptom occurs for 2 out of 3 persons, whereas with the disease sigmoid diverticulitis, the symptom occurs for 1 out of 3 persons. "vomiting" occurs with a probability of 66.7% with the disease appendicitis. "vomiting" occurs with a probability of 33.3% with the disease sigmoid diverticulitis.

Pain in the abdomen The symptom "pain in the abdomen" speaks for sigmoid diverticulitis because: With the disease sigmoid diverticulitis, the symptom "Pain in the abdomen" occurs more likely than with the disease appendicitis. With the disease "sigmoid diverticulitis," the symptom occurs for 2 out of 3 people, whereas with the disease "appendicitis," the symptom occurs for only 1 out of 3 persons. "pain in the abdomen" occurs with a probability of 66.7% with the disease sigmoid diverticulitis. "pain in the abdomen" occurs with a probability of 33.3% with the disease appendicitis. Furthermore, you have access to the patient record that records previous diagnoses of human clinicians or AI (Artificial Intelligence) clinicians. Those human and AI clinicians made their decision with the support of independent information only available to them. Thus, you know that this available information allows AI and human clinicians an accurate diagnosis with a probability of 66.7%. This means when a human or AI clinician diagnoses the patient with the disease sigmoid diverticulitis, this clinician has additional information, which speaks in 2 out of 3 cases for sigmoid diverticulitis and in 1 out of 3 cases for appendicitis. When a human or AI clinician diagnoses the patient with the disease appendicitis, this clinician has additional information, which speaks in 2 out of 3 cases for appendicitis and in 1 out of 3 cases for sigmoid diverticulitis. In the following questionnaire, we describe 52 possible situations and would like to ask you how you would diagnose the patient in the situation. We also ask you to indicate after each diagnosis how likely you think that your diagnosis is correct. After you have completed the questionnaire, you will receive 2 pounds as a base rate. If your choices match the correct answer, you will get another 2 pence for each trial. If your confidence rating lies within the range of +/- 5% of the correct probability, you will receive an additional 3 pence for each trial. The payment will be made to your account at most one week after you complete the survey.

C. Data Sets Assessment of Study One

All the data collected from the participants in the present study has been uploaded onto the OSF. Here is the anonymous link to access our data sets and the instruction for data sets. Data sets 1

D. Overall Scenarios Construction of Study Two

The table below lists the total treatments for the construction of the social conformity research project 2. Each of the following trials was repeated three times in sequence, resulting in a total of 72 trials. We illustrate the number of pieces of information, along with their diagnosis and decision accuracy within each category. For example, 1*0.65 A indicates that 1 piece of information pointed to appendicitis with 65% decision accuracy.

Trials	PrivateInfor	AI Information	Human Information	PostPro
1	1×0.55 A	3×0.65 S	1×0.55 A	81% S
2	1×0.55 S	3×0.65 A	1×0.55 S	81% A
3	1×0.55 A	3×0.70 S	1×0.55 A	89% S
4	1×0.55 S	3×0.70 A	1×0.55 S	89% A
5	1×0.55 A	3×0.85 S	1×0.55 A	99% S
6	1×0.55 S	3×0.85 A	1×0.55 S	99% A
7	1×0.65 A	3×0.55 S	1×0.65 A	65% A
8	1×0.65 S	3×0.55 A	1×0.65 S	65% S
9	1×0.70 A	3×0.55 S	1×0.70 A	75% S
10	1×0.70 S	3×0.55 A	1×0.70 S	75% A
11	1×0.80 A	3×0.70 S	1×0.80 A	56% S
12	1×0.80 S	3×0.70 A	1×0.80 S	56% A
13	1×0.55 A	3×0.65 S	1×0.55 A	81% S
14	1×0.55 S	3×0.65 A	1×0.55 S	81% A
15	1×0.55 A	3×0.70 S	1×0.55 A	89% S
16	1×0.55 S	3×0.70 A	1×0.55 S	89% A
17	1×0.55 A	3×0.85 S	1×0.55 A	99% S
18	1×0.55 S	3×0.85 A	1×0.55 S	99% A
19	1×0.65 A	3×0.55 S	1×0.65 A	65% A
20	1×0.65 S	3×0.55 A	1×0.65 S	65% S
21	1×0.70 A	3×0.55 S	1×0.70 A	75% S
22	1×0.70 S	3×0.55 A	1×0.70 S	75% A
23	1×0.80 A	3×0.70 S	1×0.80 A	56% S
24	1×0.80 S	3×0.70 A	1×0.80 S	56% A

E. Participants' Instruction of Study Two

Dear Participant, This experiment explores decision-making under uncertainty within the medical field. Your task involves imagining yourself as a clinician presented with 72 number of scenarios. These scenarios represent potential situations in a hospital setting where every Monday morning, you need to participate in a collective consultation for patients in your department and where you are required to make specific diagnoses.

Imagine yourself in the role of a clinician assessing a patient who is presenting with symptoms of either vomiting or pain in the abdomen. You must decide if the patient has one of two diseases, sigmoid diverticulitis or appendicitis. Note that a patient cannot have both diseases at the same time.

Both diseases are equally probable in the absence of any additional information about how likely each disease is given the patient's symptoms. Thus, there is a 50% chance that the patient has sigmoid diverticulitis and a 50% chance they have appendicitis. Both diseases manifest primarily through the same symptoms, but the likelihood of each disease varies depending on how strongly each symptom is associated with each disease.

For example: The symptom 'vomiting' predicts appendicitis in 4 out of 5 cases but predicts sigmoid diverticulitis in 1 out of 5 cases. Accordingly, 'vomiting' has an 80% probability of predicting

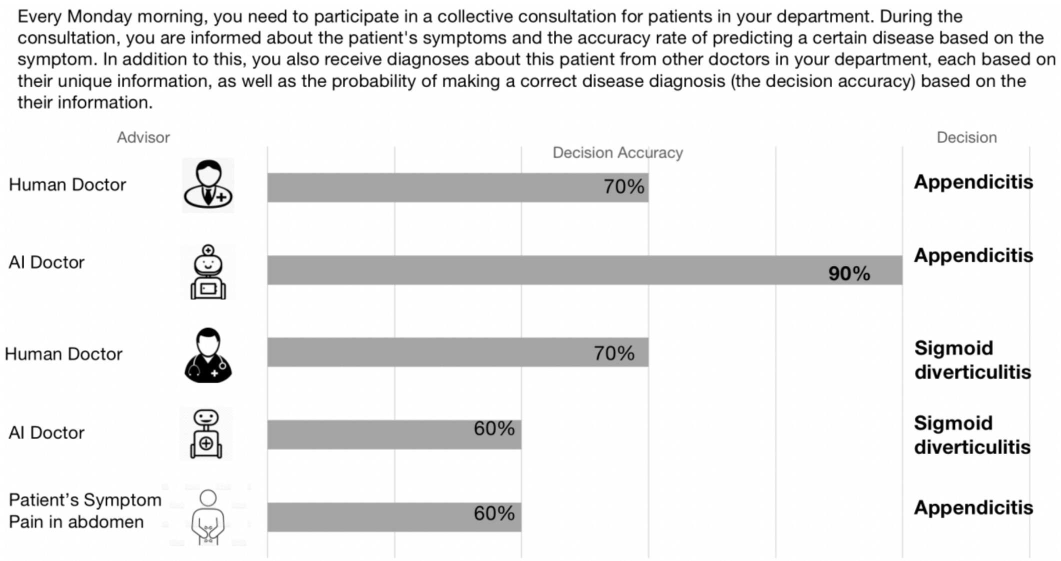

Figure 1: An example

appendicitis, but a 20% probability of predicting sigmoid diverticulitis. In this scenario, 'vomiting' is more strongly associated with appendicitis.

In each scenario, you'll receive additional information about symptoms that suggest one disease or the other. The exact probability of each symptom indicating a specific disease will be displayed via a bar chart in each scenario.

Additionally, you will review other human and/or AI doctors' diagnoses. These doctors made their diagnoses based on independent information exclusively available to them. Hence, the information available to them allows the AI and human clinicians to make an accurate diagnosis with an 'X%' probability (the so-called decision accuracy). The decision accuracy of both human and AI clinicians can vary across scenarios and will be displayed via a bar chart in each scenario.

For instance, consider the following scenario (see figure 1): You receive one piece of private information - the symptom of vomiting - which has a 60% accuracy in predicting appendicitis. Thus, appendicitis is more likely than other diseases. You also receive four pieces of advice from both human and AI doctors. The first human doctor diagnosed appendicitis with a diagnostic accuracy of 70%, while the first AI doctor's diagnosis was appendicitis with 90% accuracy. The second human doctor diagnosed sigmoid diverticulitis with a 70% decision accuracy, and the second AI doctor also diagnosed sigmoid diverticulitis with a 60% decision accuracy.

In the following questionnaire, we describe 72 possible scenarios and would like to ask you how you would diagnose the patient in the scenario. We also ask you to indicate after each diagnosis how confident you are that your diagnosis is correct.

After you have completed the questionnaire, you will receive 2 dollars as a base rate. If your choices match the correct answer, you will get another 2 cents for each scenario. If your confidence rating lies within the range of +/- 5% of the correct probability, you will receive an additional 3 cents for each scenario. The payment will be made to your account at most one week after you complete the survey.

F. Datasets of Study Two

All the data collected from the participants in the present study has been uploaded onto the Open Science Framework (OSF). Here is the anonymous link to access our data sets and the instructions for data sets: Data sets 2.
